# Dark matter phenomenology of SM and enlarged Higgs sectors extended with vector-like leptons

**DOI:** 10.1140/epjc/s10052-017-5015-2

**Published:** 2017-07-07

**Authors:** Andrei Angelescu, Giorgio Arcadi

**Affiliations:** 1 Laboratoire de Physique Théorique, Université Paris-Saclay, CNRS, 91405 Orsay, France; 2 Max Planck Institüt für Kernphysik, Saupfercheckweg 1, 69117 Heidelberg, Germany

## Abstract

We will investigate the scenario in which the Standard Model (SM) Higgs sector and its two-doublet extension (called the Two Higgs Doublet Model or 2HDM) are the “portal” for the interactions between the Standard Model and a fermionic Dark Matter (DM) candidate. The latter is the lightest stable neutral particle of a family of vector-like leptons (VLLs). We will provide an extensive overview of this scenario combining the constraints coming purely from DM phenomenology with more general constraints like Electroweak Precision Test (EWPT) as well as with collider searches. In the case that the new fermionic sector interacts with the SM Higgs sector, constraints from DM phenomenology force the new states to lie above the TeV scale. This requirement is relaxed in the case of 2HDM. Nevertheless, strong constraints coming from EWPTs and the Renormalization Group Equations (RGEs) limit the impact of VLFs on collider phenomenology.

## Introduction

Weakly Interacting Massive Particles (WIMPs) represent probably the most popular class of Dark Matter (DM) candidates. Among the features which make this kind of candidates so attractive, it is for sure worth mentioning the production mechanism. WIMP DM was indeed part of the primordial thermal bath at early stages of the history of the Universe and decoupled (freeze-out) at later stages, when the temperature was below their mass (i.e. non-relativistic decoupling), since the interactions with the SM particles were not efficient anymore with respect to the Hubble expansion rates. Under the assumption of standard cosmological history, the comoving abundance of the DM is set by a single particle physics input, namely the thermally averaged pair annihilation cross section. The experimentally favored value of DM abundance, expressed by the quantity $\Omega h^2 \approx 0.12$ [1], corresponds to a thermally averaged cross section $\langle \sigma v \rangle \sim 10^{-26}\,{\text{ cm }}^3\, {\text{ s }}^{-1}$. Interactions of this size are potentially accessible to a broad variety of search strategies, ranging from direct/indirect detection to production at colliders, making the WIMP paradigm highly testable.

From the point of view of model building, WIMP frameworks feature interactions between pairs of DM particles (in order to guarantee the cosmological stability of the DM, operators with a single DM field are in general forbidden, e.g. through a symmetry) and pair of SM states, induced by suitable mediator fields. The simplest option, in this sense, is probably represented by s-channel electrically neutral mediators, dubbed “portals”, which can couple the DM with SM fermions (see e.g. [2–4]), although couplings with the SM gauge bosons might also be feasible [5–8]. The DM relic density is thus determined via s-channel exchange of the mediator states. By simple crossing symmetry arguments these processes can be, for example, related to the rate of DM Direct Detection, induced by the t-channel interaction between the DM and the SM quarks, and to the ones of DM pair production at colliders, which can be probed mostly through mono-jet events [9–12].

Interestingly, the SM features two potential s-channel mediators, namely the *Z* and the Higgs bosons. One possible result concerns “Z-portal” DM [13] scenarios. However, they are rather contrived, since, because of gauge invariance, interactions between a SM singlet DM and the *Z* can arise only at the non-renormalizable level [14, 15]. “Higgs portal” models are instead very popular, although rather constrained [16–20], since a DM spin-0 (1), even if it is a singlet with respect to the SM gauge group, can interact with the SM Higgs doublet *H* via four-field operators connecting the bilinear $H H^\dagger$ with a DM pair and giving rise, after electroweak (EW) symmetry breaking, to an effective vertex between a DM pair and the physical Higgs field *h*.

The fermionic “Higgs portal” is instead a dimension-5 operator. Furthermore this is strongly constrained, also with respect to the scalar and vector DM cases, because of the strong direct detection rates accompanied by a velocity suppressed annihilation cross section [18, 19].

In order to couple at the renormalizable level with the *Z* and/or Higgs bosons, the fermionic DM should feature a (small) hyper- and *SU*(2) charged component. This could be realized through the mixing of a pure SM singlet and extra states with non-trivial quantum numbers under $SU(2) \times U(1)$ (see e.g. [21–24] for some constructions). The DM should then be a stable neutral state belonging to a new, non-trivial particle sector.

New chiral fermions, with mass originating from EWSB, are strongly disfavored experimentally [25]. More suitable options are instead represented by fermions belonging to a real representation or forming vector-like pairs.

In this work we will consider this last option and then extend the fermionic content of the SM with a “family” of new fields, with analogous quantum numbers as the SM leptons and the right-handed neutrinos, and with bare mass terms, which are allowed by gauge symmetry, since the new fermions are vector-like under the SM gauge group. Therefore, these fields are dubbed “vector-like leptons” (VLLs). In the absence of mixing with SM leptons, the lightest new fermionic state, if electrically neutral, constitutes a DM candidate. In this setup the DM is coupled, through Yukawa interactions, with the SM Higgs and with the *Z* and *W* bosons, featuring, in general, non-zero components charged under hypercharge and weak isospin.

This kind of scenario is, unfortunately, very strongly constrained since the Higgs and Z-boson mediate Spin Independent (SI) interactions between the DM and the nucleons, which are in increasing tension with experimental constraints. Similarly to the Higgs and Z-portal models it is possible to comply with these limits and achieve, at the same time, the correct relic density only for rather heavy DM masses or, possibly, in the presence of coannihilation processes, thus implying mass degeneracies in the new fermionic sector.

A more interesting option would consist in enlarging the mediator sector by considering two Higgs doublets (2HDM). Besides the still rather fine-tuned possibilities of s-channel resonances and coannihilations, it is possible, in this scenario, to enhance the DM annihilation cross section, marginally affecting its scattering rate on nucleons, through annihilation into extra Higgs bosons, especially the charged ones, as final states, provided that the latter are light enough. This last possibility evidences an interesting complementarity with collider searches of extra Higgs bosons. Lower limits on their masses would automatically constrain the range of viable DM masses.

LHC searches of new scalar states can be themselves influenced by the presence of new vector-like fermions since electrically charged VL fermions (which will be present in the *SU*(2) multiplet which the DM belongs to) or even color charged VL fermions (we will not consider explicitly this possibility here) can modify di-boson signal rates. For this reason 2HDM$+$VLFs models have attracted great attention in the recent times since they allowed for the interpretation of the 750 GeV diphoton excess [26–38], announced by the LHC collaboration in December 2015 [39–42], but not confirmed by the 2016 data [43, 44].

The parameters of the theory are constrained not only by the DM and collider phenomenology. The size of the couplings of the new fermions to the 125 GeV Higgs is constrained by Electro-Weak Precision Tests (EWPT). A further strong upper bound on these couplings, as well as the ones with the other Higgs states, comes from the RG running of the gauge and the quartic couplings of the scalar potential. In particular, the latter get strong negative contributions proportional to the fourth power of the Yukawa couplings of the VLLs, such that the scalar potential might be destabilized even at collider energy scales, unless new degrees of freedom are added. An important consequence of this broad variety of strong constraints is that, as will be shown below, in the parameter region corresponding to viable DM phenomenology, the collider pair production of the DM itself, through decays of the electrically neutral Higgs states, is strongly disfavored.

This work aims at an extensive overview of the phenomenology of the SM and several realizations of 2HDM extended with a sector of vector-like fermions with a stable neutral particle as lightest stable state and DM candidate.

The paper is organized as follows. We will firstly introduce, at the beginning of Sect. 2, the “family” of vector-like fermions. The remainder of the section will be dedicated to a brief overview of the SM$+$VLLs scenario. Firstly, we will briefly illustrate the general constraints coming from the modification of the Higgs signal strengths and the Electroweak Precision Tests (EWPT), and afterwards focus on the DM phenomenology. Along similar lines, an analysis for the 2HDM will then be performed in Sect. 3. After a short review of the general aspects of 2HDMs, we will perform a more detailed analysis of the constraints from EWPT and Higgs signal strengths and add to them the RGE constraints. After the analysis of the DM phenomenology, we will briefly discuss the limits/prospects, for our scenario, of collider searches. Finally, we will summarize our results in Sect. 3.7 and conclude in Sect. 4.

## Vector-like extensions of the Standard Model

In this section we will review how introducing vector-like leptons affects the SM Higgs sector. As already pointed out, the impact is mostly twofold. First of all, they generate additional loop contributions to the couplings of the Higgs boson to two photons, giving rise to deviations of the corresponding signal strength with respect to the SM prediction. In addition, the presence of vector-like leptons is typically associated with sensitive departures from experimental limits for the EW precision observables. In order to have viable values of the Higgs signal strengths and precision observables, one should impose definite relations for the Yukawa couplings and masses of the new VLLs. The same relations will hold, up to slight modifications, also in the 2HDM case.

### The vector-like “family”

In this work we will assume that the SM and, afterwards, the 2HDM Higgs sectors can be extended by “families” of vector-like fermions (VLFs). By a family we understand a set of two $SU(2)_L$ singlets and one $SU(2)_L$ doublet, belonging to a $SU(3)_c$ representation $R_c$, and with their hypercharge determined by a single parameter, *Y*. For the moment, we will keep the discussion general and later on specialize on possible DM candidates. The new fields can be schematically labeled

1 $$\begin{aligned}&\mathcal{D}_{L,R} \sim (R_c, 2, Y-1/2), \quad U^\prime _{L,R} \sim (R_c, 1, Y),\nonumber \\&D^\prime _{L,R} \sim (R_c, 1, Y-1), \end{aligned}$$

so that the couplings to the SM Higgs doublet, $H={\left( 0 \; \frac{v+h}{\sqrt{2}}\right) }^{T}$, are parametrized by the following Lagrangian:

2 $$\begin{aligned}&-\mathcal{L}_\mathrm{VLF} = y^{U_R} \overline{\mathcal{D}_L} \tilde{H} U^\prime _R + y^{U_L} \overline{U^\prime _L} \tilde{H}^\dagger \mathcal{D}_R \nonumber \\&\quad + y^{D_R} \overline{\mathcal{D}_L} H D^\prime _R + y^{D_L} \overline{D^\prime _L} H^\dagger \mathcal{D}_R \nonumber \\&\quad +M_{UD} \overline{\mathcal{D}_L} \mathcal{D}_R + M_U \overline{U^\prime _L} U^\prime _R +M_D \overline{D^\prime _L} D^\prime _R + \mathrm{h.c.}, \end{aligned}$$

where we have considered the following decomposition for the *SU*(2) doublets: $$\mathcal{D}_{L,R}\equiv \begin{pmatrix} U&D \end{pmatrix}^T_{L,R}$$.

For simplicity we will assume that all the couplings are real and that the mixing between the VLFs and the SM fermions is negligible. Later on, when specializing on DM phenomenology, we will forbid the SM fermion–VL fermion mixing through a global $\mathbb {Z}_2$ symmetry.

After electroweak symmetry breaking (EWSB), there is a mixing in the “up” ($U', U$) and “down” ($D', D$) sectors. The “up” VL fermions have charge $Q_U=Y$, while the “down” fermions have charge $Q_D=(Y-1)$. The mass matrices in the two sectors are

3 $$\begin{aligned}&\mathcal{M }_U= \begin{pmatrix} M_U &{}\quad y^{U_L} v/ \sqrt{2} \\ y^{U_R} v/ \sqrt{2} &{}\quad M_{UD} \end{pmatrix} ~,\nonumber \\&\mathcal{M}_D= \begin{pmatrix} M_D &{}\quad y^{D_L} v/ \sqrt{2} \\ y^{D_R} v/ \sqrt{2} &{}\quad M_{UD} \end{pmatrix}, \end{aligned}$$

with $v=246$ GeV, and they are bi-diagonalized as follows:

4 $$\begin{aligned}&U_L^{F} \cdot \mathcal{M}_{F} \cdot \left( U_R^{F} \right) ^\dagger = \begin{pmatrix} m_{F_1} &{} 0\\ 0 &{} m_{F_2} \end{pmatrix} ,\nonumber \\&U_L^{F} = \begin{pmatrix} c_L^{F} &{} s_L^{F} \\ -s_L^{F} &{} c_L^{F} \end{pmatrix} , \quad U_R^{F}= \begin{pmatrix} c_R^{F} &{} s_R^{F} \\ -s_R^{F} &{} c_R^{F} \end{pmatrix}, \end{aligned}$$

where the sub/superscripts $F=U,D$ distinguish between the two sectors and $c_{L/R}^F = \cos \theta _{L/R}^F$, $s_{L/R}^F = \sin \theta _{L/R}^F$. Throughout this work we will denote the lighter mass eigenstate as $F_1$. The limit where one of the singlets is decoupled, e.g. when $y_{U_R}=y_{U_L}=0$ and $M_U\rightarrow \infty$, has already been studied in detail in Ref. [45]. As we will see below the mixing structure in Eq. 3 is strongly constrained by the electroweak precision tests (EWPT) and by the Higgs couplings measurements.

### Electroweak precision tests

Extending the SM with vector-like fermions leads, in general, to the deviation of the Electroweak precision observables *S* and *T* from their respective experimental limits. Assuming negligible mixing between the SM and the vector-like fermions, the limits on *S* and *T* can be directly translated into limits on the Yukawa couplings and masses of the new fermions; in the limit in which the former go to zero, no constraints from EWPT apply.

Sizable values of the Yukawa couplings of the VLFs can nevertheless be obtained while still complying with the limits on the *T* parameter by relying (at least approximately) on a custodial limit:

5 $$\begin{aligned} M_D=M_U, \qquad y^{U_L}=y^{D_L}, \qquad y^{U_R}=y^{D_R}, \end{aligned}$$

which is equivalent to imposing equal mass matrices in the isospin-up and isospin-down sectors. Clearly, the custodial limit can be achieved only by considering “full families” of VLFs, i.e. a corresponding SU(2) singlet for each of the components of the doublet, as done in this work.

**Fig. 1:**
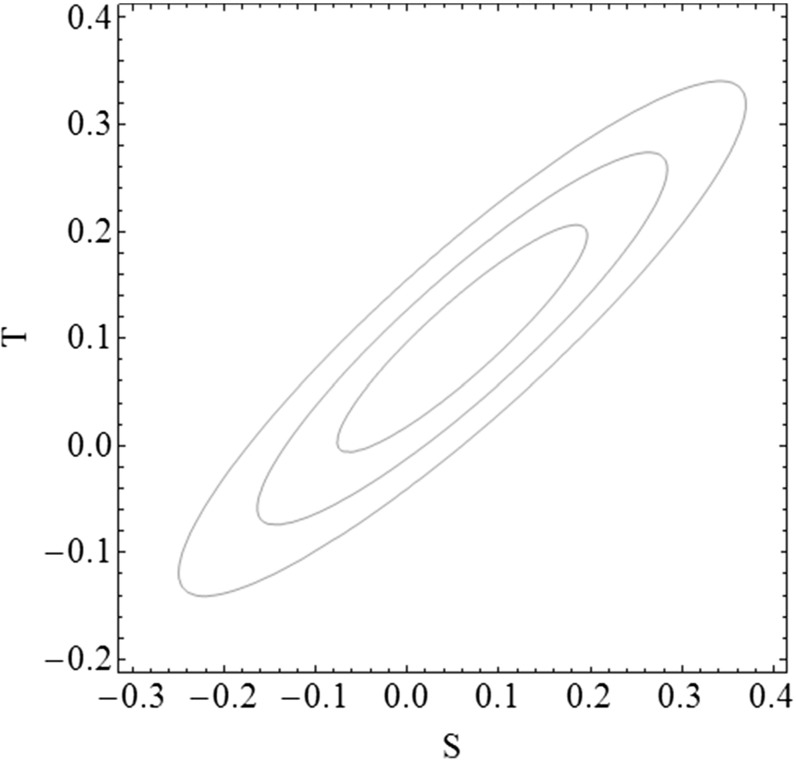
Allowed values of S and T [46] at, from the innermost to the outermost ellipse, 68, 95.5 and 99.7% confidence level (CL)

On the contrary, there is no symmetry protecting the *S* parameter, which means that, in some cases, it will impose more relevant constraints than the *T* parameter. The constraints on *S* can be nevertheless partially relaxed by taking advantage of the correlation among the *S* and *T* parameters, illustrated in Fig. 1, by allowing for a small deviation from the custodial limit, i.e. $T \gtrsim 0$.

### Higgs couplings

We now turn to the second constraint coming from the Higgs couplings measurements. In the presence of vector-like fermions, its couplings to gauge bosons receive additional contributions, originating from triangle loops in which the new fermions are exchanged. No new decay channels into VLFs are instead present since, because of constraints from direct searches at colliders, the VLFs should be heavier than the SM Higgs.

The SM Higgs loop-induced partial decay widths into massless gauge bosons, $\Gamma _{h \mathrm {VV}},\, \mathrm {V}=g,\gamma$, can be schematically expressed as $\Gamma _{h\mathrm {VV}} \propto |\mathcal {A}_\mathrm{SM}^{h\mathrm {VV}}+\mathcal {A}_\mathrm{VLF}^{h\mathrm {VV}}|^2$, where $\mathcal {A}_\mathrm{SM}^{h\mathrm {VV}}$ and $\mathcal {A}_\mathrm{VLF}^{h\mathrm {VV}}$ represent the amplitudes associated, respectively, with the SM and VLF contributions. Throughout this work we will only consider the case of a family of color-neutral VLFs ($R_c=1$); as a consequence the new physics sector influences mostly $\Gamma _{h\gamma \gamma }$ and therefore the $h\rightarrow \gamma \gamma$ signal strength, $\mu _{\gamma \gamma }$.^1^ The corresponding amplitude is given by

6 $$\begin{aligned} \mathcal{A}^{h\gamma \gamma }_\mathrm{VLF}= \sum _{\begin{array}{c} F=U,D \\ i=1,2 \end{array}} Q_F^2 \frac{v (\mathcal {C}_F)_{ii}}{m_{F_i}}A_{1/2}^h (\tau ^h_{F_i}), \end{aligned}$$

where $\tau ^h_{F_i}=\frac{m_h^2}{4 m_{F_i}^2}$, while $A_{1/2}^h$ is a loop form factor whose definition is given e.g. in [49]. The matrix $\mathcal {C}_F$ is defined as

7 $$\begin{aligned} \mathcal {C}_F=U_F^L \cdot \mathcal {Y}_F \cdot (U_F^R)^{\dagger },\quad \mathcal {Y}_F=\partial _v \mathcal {M}_F= \frac{1}{\sqrt{2}} \begin{pmatrix} 0 &{} y_h^{F_L}\\ y_h^{F_R} &{} 0 \end{pmatrix}. \end{aligned}$$

For a 125 GeV Higgs we can reliably approximate the loop function $A_{1/2}^h (\tau )$ with its asymptotic value, for $A_{1/2}^h (0) = 4/3$, such that the expression (6) simplifies to

8 $$\begin{aligned} \mathcal{A}^{h\gamma \gamma }_\mathrm{VLF}=A_{1/2}^{h} (0) \sum _{F=U,D} \frac{-2 v^2 y_h^{F_L} y_h^{F_R}}{2 M_F M_{UD} - v^2 y_h^{F_L} y_h^{F_R}}. \end{aligned}$$

Experimental measurements do not exhibit statistically relevant deviations of $\mu _{\gamma \gamma }$ from the SM prediction [47, 48], which implies essentially two possibilities: $\mathcal {A}_\mathrm{VLL}^{h \gamma \gamma } \simeq 0$ or $\mathcal {A}_\mathrm{VLL}^{h \gamma \gamma } \simeq -2 \mathcal {A}_\mathrm{SM}^{h \gamma \gamma }$. As evident from Eq. (8), the first possibility is easily realized by setting to zero one of the $y_h^{F_{L,R}}$ couplings.^2^ The other is instead more complicated to realize. Assuming $Y=0$ (as will be done for the rest of the paper), such that only *D*-type states contribute to $\mu _{\gamma \gamma }$, and setting for simplicity $M_D=M_{UD}$ and $y_h^{D_L}=-y_h^{D_R}=y_h^D$, which implies that the two mass eigenstates will have the same mass $m_D$, the relation to impose becomes

9 $$\begin{aligned} \mathcal {A}_\mathrm{VLL}^{h \gamma \gamma }= \frac{4}{3} {\left( \frac{y_h^D v}{m_D}\right) }^2 \simeq -2 \mathcal {A}_\mathrm{SM}^{h \gamma \gamma } \simeq 13, \end{aligned}$$

which is impossible to satisfy since $y_h^D v / m_D$ is smaller than 2 (or equal, for $M_D=M_{UD}=0$).^3^ Unless stated otherwise, we will always consider, for both the SM and the 2HDM cases, an assignation of the Yukawa couplings of the VLFs such that $\mathcal {A}_\mathrm{VLL}^{h\gamma \gamma }=0$.

### DM phenomenology

A DM candidate is introduced, in our setup, by considering a “family” of vector leptons coupled with the SM Higgs doublet according to the following lagrangian:

10 $$\begin{aligned}&-\mathcal{L}_{VLL}= y_h^{N_R} \overline{L}_L \tilde{H} N_R^\prime + y_h^{N_L} \overline{N}^\prime _L \tilde{H}^\dagger L_R \nonumber \\&\quad +y_h^{E_R} \overline{L}_L H E^\prime _R + y_h^{E_L} \overline{E}^\prime _L H^\dagger L_R \nonumber \\&\quad +M_L \overline{L}_L L_R + M_N \overline{N}^\prime _L N^\prime _R\nonumber \\&\quad +M_E \overline{E}^\prime _L E^\prime _R + \mathrm {h.c.}. \end{aligned}$$

To guarantee the stability of the DM candidate, we impose a global $\mathbb {Z}_2$ symmetry under which the vector-like leptons are odd and the SM is even (a supersymmetric analogue is the well-known R-parity). After EW symmetry breaking a mixing between the vector-like fermions is generated, as described by the following mass matrices:

11 $$\begin{aligned}&\mathcal {M}_N = \left( \begin{array}{cc} M_N &{} v' y_h^{N_L} \\ v' y_h^{N_R} &{} M_L \end{array}\right) , \end{aligned}$$

12 $$\begin{aligned}&\mathcal {M}_L = \left( \begin{array}{cc} M_E &{} v' y_h^{E_L} \\ v' y_h^{E_R} &{} M_L \end{array}\right) . \end{aligned}$$

where $v' = v/\sqrt{2} \simeq 174$ GeV. Note that the $\mathbb {Z}_2$ symmetry prevents mixing between the VLLs and the SM fermions. In order to pass from the interaction to the mass basis one has to bidiagonalize the above matrices as

13 $$\begin{aligned}&U_L^N \cdot \mathcal {M}_N \cdot ( U_R^N )^{\dagger } = \mathrm {diag} (m_{N_1}, m_{N_2}), \nonumber \\&U_L^E \cdot \mathcal {M}_E \cdot ( U_R^E )^{\dagger } = \mathrm {diag} (m_{E_1}, m_{E_2}), \end{aligned}$$

with the unitary matrices $U_{L,R}^{F}$, $F=N,E$ written explicitly as

$$\begin{aligned} U_{L,R}^F=\left( \begin{array}{cc} \cos \theta _{L,R}^F &{} \sin \theta _{L,R}^F \nonumber \\ -\sin \theta _{L,R}^F &{} \cos \theta _{L,R}^F \end{array} \right) , \end{aligned}$$

where

14 $$\begin{aligned} \tan 2 \theta _L^N =\frac{2 \sqrt{2} v \left( M_L y_h^{N_L}+ M_N y_h^{N_R}\right) }{2 M_L^2-2 M_N^2 -v^2 \left( |y_h^{N_L}|^2-|y_h^{N_R}|^2\right) },\nonumber \\ \tan 2 \theta _R^N =\frac{2 \sqrt{2} v \left( M_N y_h^{N_L}+ M_L y_h^{N_R}\right) }{2 M_L^2-2 M_N^2 +v^2 \left( |y_h^{N_L}|^2-|y_h^{N_R}|^2\right) }. \end{aligned}$$

The corresponding expressions for $\theta _{L,R}^E$ can be found from the ones above by replacing $M_N \rightarrow M_E$ and $y_h^{N_{L,R}} \rightarrow y_h^{E_{L,R}}$.

The DM candidate $N_1$ (i.e. the lighter VL neutrino) is in general a mixture of the *SU*(2) singlet (with null hypercharge) $N_{L,R}^{'}$ and doublet $N_{L,R}$. As a consequence it is coupled with the Higgs scalar *h* as well as with the SM gauge bosons $W^{\pm }$ and *Z*. These couplings are given by

15 $$\begin{aligned}&y_{h N_1 N_1}= \frac{\cos \theta _N^L \sin \theta _N^R y_h^{N_L}+\cos \theta _N^R \sin \theta _N^L y_h^{N_R}}{\sqrt{2}}, \nonumber \\&y_{V,Z N_1 N_1}=\frac{g}{4 \cos \theta _W}( \sin ^2 \theta _L^N + \sin ^2 \theta _R^N ), \nonumber \\&y_{A,Z N_1 N_1}=\frac{g}{4 \cos \theta _W}( \sin ^2 \theta _L^N - \sin ^2 \theta _R^N ), \nonumber \\&y_{V, W N_1 E_1}=\frac{g}{2 \sqrt{2}} ( \sin \theta _L^N \sin \theta _L^E + \sin \theta _R^N \sin \theta _R^E ), \nonumber \\&y_{A, W N_1 E_1}=\frac{g}{2 \sqrt{2}} ( \sin \theta _L^N \sin \theta _L^E - \sin \theta _R^N \sin \theta _R^E ), \end{aligned}$$

where, for convenience, we have expressed the couplings with the *Z* and *W* bosons in terms of vectorial and axial combinations.

The DM relic density can be determined through the WIMP paradigm as a function of the DM thermally averaged pair annihilation cross section, formally defined (excluding coannihilations) as [50]:

16 $$\begin{aligned} \langle \sigma v \rangle&=\frac{1}{8 m_{N_1}^4 T K_2^2(m_{N_1}/T)}\nonumber \\&\,\quad \times \int _{4 m_{N_1}^2}^{\infty } \mathrm{d}s \sigma (s) (s-4 m_{N_1}^2)\sqrt{s} K_1(\sqrt{s}/T), \end{aligned}$$

which is in turn a function of the couplings reported in Eq. (15). The possible DM annihilation processes consist in annihilations into SM fermions pairs, induced by s-channel exchange of the *h* and *Z* bosons, and into $W^+ W^-$, *ZZ*, *Zh*, and *hh*, induced also by t-channel exchange of the neutral states $N_{1,2}$ ($E_{1,2}$ for the $W^+ W^-$ final state). In order to precisely determine the DM relic density we have numerically computed (16) through the package micromegas [51]. We will nevertheless provide some simple approximations to facilitate the comprehension of the relationship between the DM relic density and the relevant parameters of the theory, obtained by the conventional velocity expansion [52] $\langle \sigma v \rangle \approx a + 2 b/x$ (using $\sigma v \approx a+b v^2/3, \langle v^2 \rangle =6/x)$ and taking only, if non-vanishing, the leading, s-wave, coefficient *a*.^4^

In the case of annihilation into $\bar{f} f$ final states, the only non-vanishing contribution in the $v \rightarrow 0$ limit is the one associated to the s-channel *Z*-exchange:

17 $$\begin{aligned}&\langle \sigma v \rangle _{ff} \approx \frac{m_{N_1}^2}{8 \pi } \frac{g^2 m_{N_1}^2}{\pi ((4 m_{N_1}^2-m_Z^2)^2+m_Z^2 \Gamma _Z^2)}\nonumber \\&\quad \times \sum n_c^f (|V_f|^2+|A_f|^2) |y_{V,Z N_1 N_1}|^2, \end{aligned}$$

where $V_f$ and $A_f$ are the vectorial and axial couplings of the *Z*-boson and the SM fermions:

18 $$\begin{aligned} V_f=\frac{g}{2 c_W} (-2 q_f s_W^2 +T^3_f),\,\,\,\,\,A_f=\frac{g}{2 c_W} T^3_f, \end{aligned}$$

while $n_c^f$ is the color factor and $s_W=\sin \theta _W$ and $c_W=\cos \theta _W$. The cross sections of the other relevant final states can be instead estimated as^5^:

19 $$\begin{aligned}&\langle \sigma v \rangle _{W^+ W^-} \approx \frac{g^4 t_W}{16 \pi m_W^2} ((\sin \theta _L^N)^2+(\sin \theta _R^N)^2)^2 \nonumber \\&\quad +\frac{g^4}{64}\left( \frac{1}{2 \pi } ((\sin \theta _L^N \sin \theta _L^E)^2+(\sin \theta _R^N \sin \theta _R^E)^2)^2\right. \nonumber \\&\quad \times \,\left. \frac{m_{N_1}^2}{(m_{N_1}^2+m_{E_1}^2)^2}+\frac{2}{\pi }((\sin \theta _L^N \sin \theta _L^E)^2\right. \nonumber \\&\quad \left. -(\sin \theta _R^N \sin \theta _R^E)^2)^2 \frac{m_{N_1}^4}{m_W^4}\frac{m_{E_1}^2}{(m_{N_1}^2+m_{E_1}^2)^2}\right) , \end{aligned}$$

Here $t_W=\tan \theta _W$. We have

20 $$\begin{aligned}&\langle \sigma v \rangle _{ZZ} \approx \frac{g^4}{32 \pi c_W^4 m_Z^2}\nonumber \\&\quad \times \left[ \frac{m_Z^2}{4 m_{N_1}^2} \left( \left| (\sin \theta _L^N)^2+(\sin \theta _R^N)^2\right| ^4\right. \right. \nonumber \\&\quad \left. \left. +\left| (\sin \theta _L^N)^2-(\sin \theta _R^N)^2\right| ^4\right) \right. \nonumber \\&\quad \left. +2 \left| (\sin \theta _L^N)^2+(\sin \theta _R^N)^2\right| ^2\right. \nonumber \\&\quad \times \left. \left| (\sin \theta _L^N)^2-(\sin \theta _R^N)^2\right| ^2\right] , \end{aligned}$$

and

21 $$\begin{aligned} \langle \sigma v \rangle _{Zh} \approx \frac{g^2}{4 \pi v^2} |y_{V, Z N_1 N_1}|^2 \frac{m_Z^2}{m_{N_1}^2}. \end{aligned}$$

The achievement of the correct relic density through DM annihilations can be potentially in tension with limits from direct detection experiments. Indeed, DM interactions with SM quarks, mediated by t-channel exchange of *Z* and *h* bosons, induce both Spin Independent (SI) and Spin Dependent (SD) scattering processes of the DM with nuclei of target detectors.

The corresponding cross sections, focusing for simplicity on the scattering on protons, are given by

22 $$\begin{aligned}&\sigma _{N_1 p, Z}^{SI}=\frac{\mu _{N_1}^2}{\pi }\frac{1}{m_Z^4}|y_{V,Z N_1 N_1}|^2 \nonumber \\&\quad \times {\left[ \left( 1+\frac{Z}{A}\right) V_u + \left( 2-\frac{Z}{A}\right) V_d\right] }^2\nonumber \\&\sigma _{N_1 p, h}^{SI}=\frac{\mu _{N_1}^2}{\pi }\frac{m_p^2}{v^2}\frac{1}{m_h^4}\nonumber \\&\quad \times \left| y_{h N_1 N_1} \left( \sum _{q=u,d,s}f_q +\frac{2}{27}f_{TG} \sum _{q=c,b,t}\right) \right| ^2, \end{aligned}$$

23 $$\begin{aligned}&\sigma _{N_1 p}^{SD}=\frac{3\mu _{N_1}^2}{\pi m_Z^4} |y_{A, Z N_1 N_1}|^2 \big [ A_u (\Delta _u^p S_p^A+\Delta _u^n S_n^A) \nonumber \\&\quad +A_d ((\Delta _d^p+\Delta _s^p)S_p^A +(\Delta _d^n+\Delta _s^n)S_n^A)\big ]^2 \frac{1}{(S_p^A+S_n^A)^2}. \end{aligned}$$

In the expressions above, $\mu _{N_1}=\frac{m_p m_{N_1}}{m_p+m_{N_1}}$, $f_q, f_{TG},\Delta _q^{p,n}$ are nucleon form factors, while $S_p^A$ and $S_n^A$ are the contributions of the proton and neutron to the spin of the nucleus *A*. We have used the values reported in [54].

Among these contributions, the most important one is represented by the SI cross section from *Z*-mediated interactions. This allows us to estimate the SI cross section as

24 $$\begin{aligned} \sigma _{N_1 p}^\mathrm{SI} \approx 2 \times 10^{-39}\,{\text{ cm }}^2 {( \sin ^2 \theta _L^N+\sin ^2 \theta _R^N)}^2. \end{aligned}$$

In order to comply with the stringent limits by the LUX experiment [55] which impose, for DM masses of the order of few hundreds GeV, a cross section of the order of $10^{-45}\,{\text{ cm }}^2$, we need to require $\sqrt{\sin ^2 \theta _L^N+\sin ^2 \theta _R^N} \sim 10^{-(1 \div 2)}$.

We have computed the main DM observables, i.e. relic density and SI scattering cross section, for a sample of model points generating by scanning on the parameters $(y_h^{N_{L,R}},y_h^{E_{L}},M_N,M_E,M_L)$, while we set $y_h^{E_R}=0$ in order to achieve $\mathcal {A}_{NP}^{h\gamma \gamma }=0$, over the following range:

25 $$\begin{aligned}&y_h^{N_{L,R}} \in [10^{-3},1], \nonumber \\&y_h^{E_{L}} \in [5 \times 10^{-3},3], \\&M_N \in [100\,\text{ GeV }, 5\,\text{ TeV }], \nonumber \\&M_E=M_L \in [300\,\text{ GeV }, 5\,\text{ TeV }],\nonumber \end{aligned}$$

with the additional requirement of not exceeding the limits from EWPT.

**Fig. 2:**
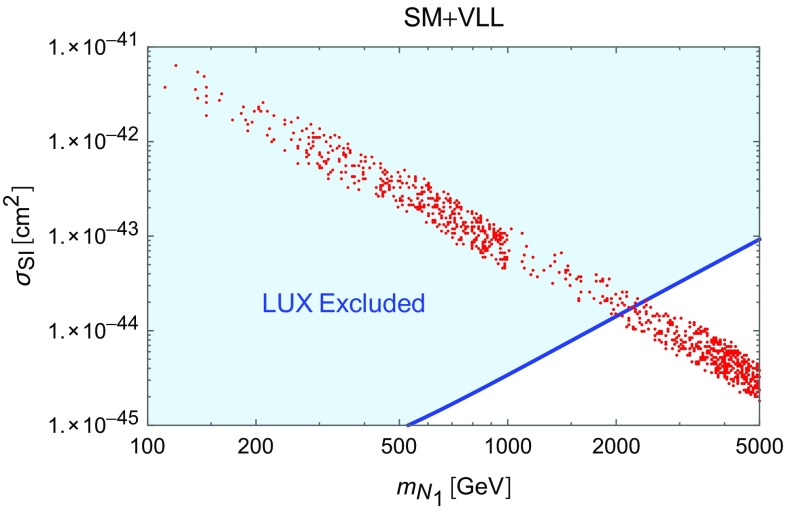
Model points satisfying EWPT and Higgs width constraints and providing the correct DM relic density (see main text for clarification) reported in the bidimensional plane $(m_{N_1},\sigma _p^\mathrm{SI})$. The *blue region* is excluded by current constraints from DM direct detection

The results of our analysis are reported in Fig. 2. The figure shows the set of points featuring the correct DM relic density in the bidimensional plane $(m_{N_1},\sigma _\mathrm{SI})$. As evident, the very strong constraints from the *Z*-mediated DM scattering on nucleons rule out the parameter space corresponding to thermal DM unless its mass is approximately above 2 TeV. This result is very similar to what is obtained in the generic scenario dubbed *Z*-portal [13], in which the SM *Z* boson mediates the interactions between the SM states and a Dirac fermion DM candidate. Notice that in our parameter scan we have anyway considered DM masses heavier than 100 GeV and a sizable mass splitting between the DM and the lightest electrically charged fermion $E_1$. If these requirements are dropped, one could achieve an enhanced DM annihilation cross section at the Higgs “pole”, i.e. $m_{N_1} \simeq m_h/2$, or through coannihilations (we will discuss this scenario in the next section in the context of the 2 Higgs doublet model), eventually relaxing the tensions with DD. This possibility has been considered e.g. in [56] in a similar model (notice that in this reference the VL neutrinos have also Majorana mass terms and their interactions with the *Z* are weaker than in the model discussed here), as the one discussed here, but focused on the case of rather light VLLs enhancing the decay branching fraction of the SM Higgs to diphotons (we have instead considered the case in which this coincides with the SM expectation).

### Vacuum stability

As will be discussed in greater detail in the next section, the presence of vector-like fermions has a very strong impact on the behavior of the theory with respect to radiative corrections. The RG evolution of the parameters of the scalar potential typically suffers the strongest influence from the introduced new physics.

As is well known, the stability of the EW vacuum depends on the sign of the quartic coupling $\lambda$ of the Higgs potential. This parameter, positive at the electroweak scale, is driven towards negative values, at high energy scales, by radiative corrections mostly relying on the Yukawa coupling of the top quark. Detailed studies, like e.g. [57] have shown that the EW vacuum is stable up to energies close to the Planck scale. A quantitative determination is nevertheless extremely sensitive to the value of the mass of the top quark.

The presence of vector-like fermions tends to make steeper the decrease of $\lambda$ at high energy as can be seen by the one-loop $\beta$ function:

26 $$\begin{aligned}&\beta _{\lambda }=\frac{1}{16 \pi ^2} [\beta _{\lambda ,\mathrm SM}+4 \lambda ((y_h^{N_L})^2+(y_h^{N_R})^2+(y_h^{E_L})^2+(y_h^{E_R})^2)\nonumber \\&~~~~~~\quad -4 ((y_h^{N_L})^4+(y_h^{N_R})^4+(y_h^{E_L})^4+(y_h^{E_R})^4)] \end{aligned}$$

where $\beta _{\lambda ,\mathrm SM}$ accounts for the SM contribution.

We have then checked the stability of the EW vacuum in the presence of the new vector-like fermions by solving the coupled RGE’s of the Higgs quartic couplings and of relevant parameters as the yukawa couplings of the VLF and of the top quark and the gauge couplings.^6^ For simplicity we have assumed that all the new particles lie at a same scale $m_F=400\,\text{ GeV }$ and that their Yukawa couplings are zero below this scale. As discussed in the previous subsection, the coupling $y_h^{N_L}$ is constrained, by DM phenomenology, to be very small, below $10^{-2}$. We also recall that we customarily assume $y_h^{N_R}=y_h^{E_R}=0$ to automatically comply with the constraints on the Higgs signal strength. In this simplified picture the vacuum stability depends, besides the SM inputs, on just one new parameter, i.e. $y_h^{E_L}$.

**Fig. 3:**
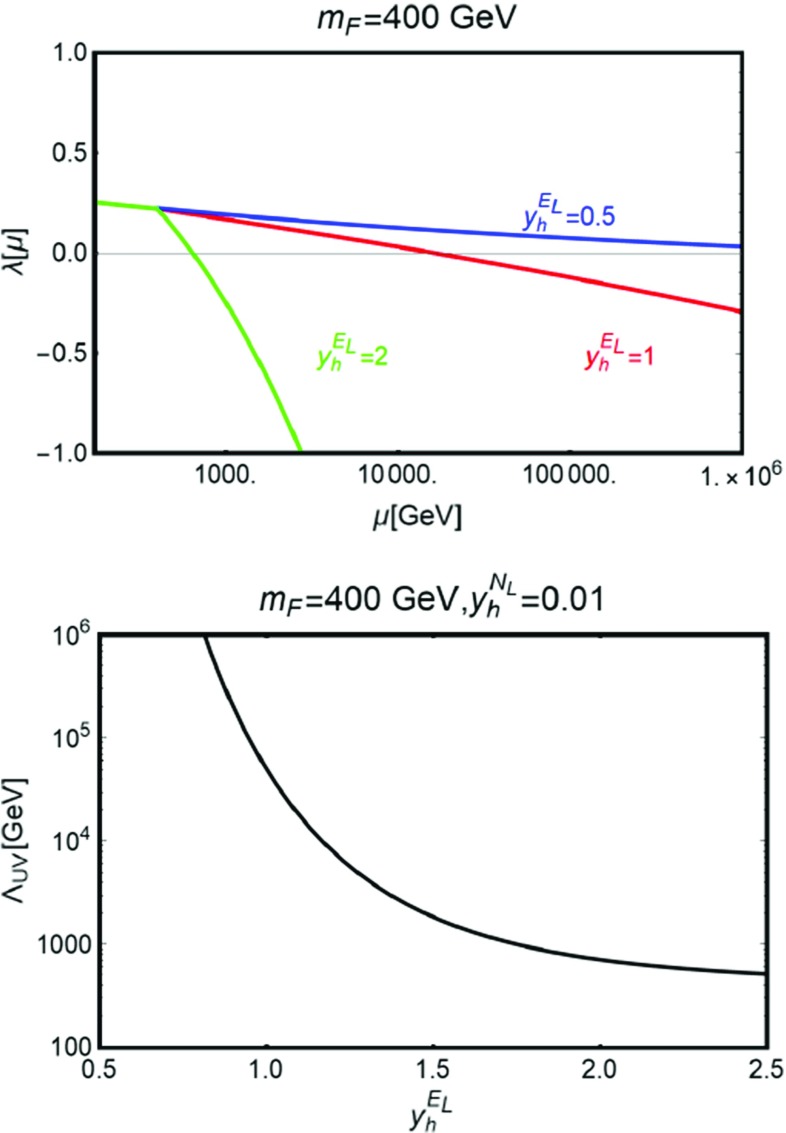
*Top panel* evolution of the Higgs quartic coupling $\lambda$ with the energy scale $\mu$ for three assignations, i.e. 0.5, 1 and 2, of the Yukawa coupling $y_h^{E_L}$. The VLL have been assumed to be at a scale $m_F=400\,\text{ GeV }$ while the neutral Yukawa coupling $y_h^{N_L}$ has been set to 0.01. The other two Yukawa couplings $y_h^{N_R},y_h^{E_R}$ have been set to zero in order to have a SM-like diphoton rate for the Higgs boson. *Bottom panel* evolution of the scale $\Lambda _\mathrm{UV}$, defined by $\lambda (\Lambda _\mathrm{UV})=-0.07$ with the coupling $y_h^{E_L}$. The other parameters have been set as in the *upper panel*

The behavior of the Higgs quartic parameter with the energy is shown, for some assignations of $y_h^{E_L}$, in the first panel of Fig. 3. As is evident, values of $y_h^{E_L}$ are equal to or greater than one correspond to a fast drop of $\lambda$. For $y_h^{E_L}=2$ we notice indeed that the Higgs quartic coupling becomes negative in proximity of the energy threshold corresponding to the mass of the VLF. Our results have been made more quantitative in the second panel of Fig. 3. Here we have indeed defined the stability scale $\Lambda _\mathrm{UV}$, adopting the criterion $\lambda (\Lambda _\mathrm{UV})=-0.07$ [58]. The energy scale $\Lambda _\mathrm{UV}$ is interpreted as the scale below which new degrees of freedom should be added in order to have stability of the EW up to high scales. As will be further remarked along the paper, building a UV complete model is not in the purposes of present work; as a consequence we will implicitly adopt the minimal requirement that $\Lambda _\mathrm{UV}$ lies above the energy scales accessible to collider studies, namely few TeVs, so that our model provides a reliable low energy description of the relevant phenomenology.

## Two Higgs doublet models

Let us now move to the case of 2HDM scenarios. We will summarize below their most salient features and fix, as well, the notation. For a more extensive review we refer instead, for example, to [59].

In this work we have considered the following, CP-conserving, scalar potential:

27 $$\begin{aligned}&V(H_1,H_2) = m_{11}^2 H_1^\dagger H_1+ m_{22}^2 H_2^\dagger H_2\nonumber \\&\quad - m_{12}^2 (H_1^\dagger H_2 + \mathrm{h.c.} ) +\frac{\lambda _1}{2} ( H_1^\dagger H_1 )^2\nonumber \\&\quad +\frac{\lambda _2}{2} ( H_2^\dagger H_2 )^2 \nonumber \\&\quad + \lambda _3 (H_1^\dagger H_1 )(H_2^\dagger H_2 ) + \lambda _4 (H_1^\dagger H_2 )(H_2^\dagger H_1 )\nonumber \\&\quad +\frac{\lambda _5}{2}[ (H_1^\dagger H_2 )^2 + \mathrm{h.c.} ], \end{aligned}$$

where two doublets are defined by

28 $$\begin{aligned} H_i= \begin{pmatrix} \phi _i^+ \\ (v_i+\rho _i +i \eta _i)/\sqrt{2} \end{pmatrix}, \qquad i=1,2, \end{aligned}$$

where, as usual, $v_2/v_1= \tan \beta \equiv t_{\beta }$. The most general scalar potential would feature two additional quartic couplings $\lambda _6$, $\lambda _7$ which have been, for simplicity, set to zero (this can be achieved by imposing a discrete symmetry [60]).

**Fig. 4:**
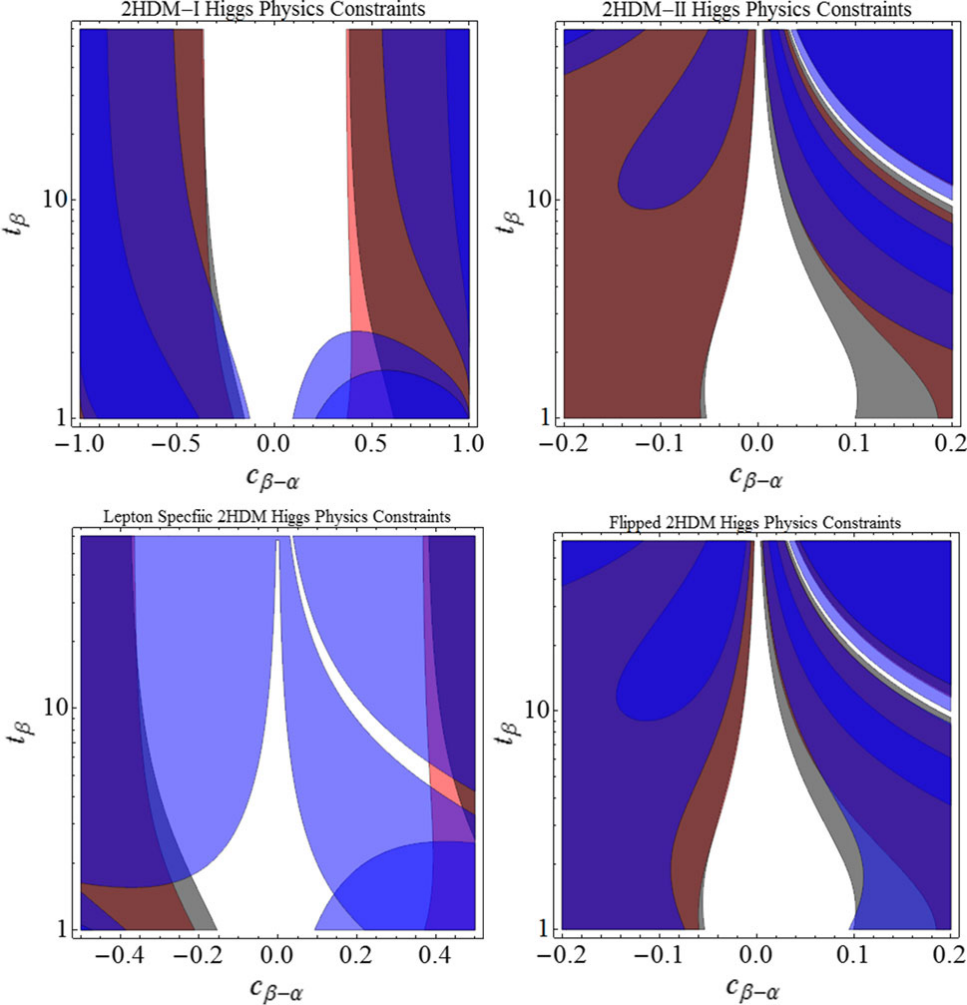
Constraints in the $\left( c_{\beta -\alpha }, t_{\beta }\right)$ plane on the four types of flavor-conserving 2HDMs, coming from Higgs signal strength measurements [47, 48]. The signal strengths we have considered are $\mu _{\gamma \gamma }$ (*red*), $\mu _{ZZ,WW}$ (*gray*), and $\mu _{bb,\tau \tau }$ (*blue*)

The spectrum of physical states is constituted by two CP-even neutral states, *h*, identified with the 125 GeV Higgs, and *H*, the CP-odd Higgs *A* and finally the charged Higgs $H^{\pm }$. The transition from the interaction basis $(H_1,H_2)^T$ to the mass basis $(h,H,A,H^{\pm })$ depends on two mixing angles, $\alpha$ and $\beta$. Throughout this work we will assume the so-called alignment limit, i.e. $\alpha \simeq \beta -\pi /2$. This is a reasonable assumption since, in most scenarios, as also shown in Fig. 4, only small deviations from the alignment limit are experimentally allowed. In this limit, the *h* boson becomes completely SM-like. A second relevant implication is that the couplings of the second CP Higgs *H* with *W* and *Z* bosons are zero at tree level, being proportional to $\cos (\beta -\alpha )$ (analogous tree-level couplings for the *A* boson are forbidden by CP conservation). For a more detailed treatment of the alignment limit, we refer the reader to e.g. Refs. [61–65].

The quartic couplings of the scalar potential (27) can be expressed as a function of the masses of the physical states as

29 $$\begin{aligned} \lambda _1&= \frac{1}{v^2} [ m_h^2 + ( m_H^2 - M^2 ) t_{\beta }^2 ], \end{aligned}$$

30 $$\begin{aligned} \lambda _2&= \frac{1}{v^2} [ m_h^2 + ( m_H^2 - M^2 ) t_{\beta }^{-2} ], \end{aligned}$$

31 $$\begin{aligned} \lambda _3&= \frac{1}{v^2} [ m_h^2 + 2 m_{H^{\pm }}^2 - ( m_H^2 + M^2 ) ], \end{aligned}$$

32 $$\begin{aligned} \lambda _4&= \frac{1}{v^2} [ M^2 + m_A^2 - 2 m_{H^{\pm }} ], \end{aligned}$$

33 $$\begin{aligned} \lambda _5&= \frac{1}{v^2} [ M^2 - m_A^2 ], \end{aligned}$$

where $M \equiv m_{12}/(s_{\beta } c_{\beta })$. Unitarity and boundedness from below of the scalar potential impose constraints on the value of the couplings $\lambda _{i=1,5}$ [59, 66] which, through Eq. 29, are translated into bounds on the physical masses. In particular these bounds imply that it is not possible to assign their values independently one from each other. All these bounds can be found, for example, in Refs. [66, 67], but, for completeness, we will report them below. For the scalar potential to be bounded from below, the quartics must satisfy

34 $$\begin{aligned} \lambda _{1,2}> 0, \; \lambda _3> -\sqrt{\lambda _1\lambda _2}, \; \mathrm{and} \; \lambda _3 + \lambda _4 - \left| \lambda _5\right| > -\sqrt{\lambda _1\lambda _2},\qquad \end{aligned}$$

while s-wave tree-level unitarity imposes the requirement

35 $$\begin{aligned} \left| a_{\pm } \right| , \left| b_{\pm } \right| , \left| c_{\pm } \right| , \left| f_{\pm } \right| , \left| e_{1,2} \right| , \left| f_1 \right| , \left| p_1 \right| < 8\pi , \end{aligned}$$

where

36 $$\begin{aligned} a_{\pm }&= \frac{3}{2}(\lambda _1 + \lambda _2) \pm \sqrt{\frac{9}{4}(\lambda _1-\lambda _2)^2 + (2\lambda _3 + \lambda _4)^2}, \nonumber \\ b_{\pm }&= \frac{1}{2}(\lambda _1 + \lambda _2) \pm \sqrt{(\lambda _1-\lambda _2)^2 + 4\lambda _4^2}, \nonumber \\ c_{\pm }&= \frac{1}{2}(\lambda _1 + \lambda _2) \pm \sqrt{(\lambda _1-\lambda _2)^2 + 4\lambda _5^2}, \nonumber \\ e_1&= \lambda _3 + 2\lambda _4 - 3\lambda _5, \quad e_2 = \lambda _3 - \lambda _5, \nonumber \\ f_+&= \lambda _3 + 2\lambda _4 + 3\lambda _5, \!\quad f_- = \lambda _3 + \lambda _5, \nonumber \\ f_1&= \lambda _3 + \lambda _4, \quad p_1 = \lambda _3 - \lambda _4. \end{aligned}$$

Vacuum stability finally requires [68]:

37 $$\begin{aligned} m_{12}^2 \left( m_{11}^2-m_{22}^2 \sqrt{\lambda _1/\lambda _2}\right) \left( \tan \beta -\root 4 \of {\lambda _1/\lambda _2}\right) >0 \end{aligned}$$

**Table 1:** Couplings of the Higgses to the SM fermions as a function of the angles $\alpha$ and $\beta$ and in the alignment limit where $(\beta -\alpha ) \rightarrow \pi /2$

	Type I	Type II	Lepton-specific	Flipped
$\xi _h^u$	$c_{\alpha }/ s_{\beta } \rightarrow 1$	$c_{\alpha }/ s_{\beta } \rightarrow 1$	$c_{\alpha }/ s_{\beta } \rightarrow 1$	$c_{\alpha }/ s_{\beta }\rightarrow 1$
$\xi _h^d$	$c_{\alpha }/ s_{\beta } \rightarrow 1$	$-s_{\alpha }/ c_{\beta } \rightarrow 1$	$c_{\alpha }/ s_{\beta } \rightarrow 1$	$-s_{\alpha }/ c_{\beta } \rightarrow 1$
$\xi _h^l$	$c_{\alpha }/ s_{\beta } \rightarrow 1$	$-s_{\alpha }/ c_{\beta } \rightarrow 1$	$-s_{\alpha }/ c_{\beta } \rightarrow 1$	$c_{\alpha }/ s_{\beta } \rightarrow 1$
$\xi _H^u$	$s_{\alpha }/ s_{\beta } \rightarrow -t_{\beta }^{-1}$	$s_{\alpha }/ s_{\beta } \rightarrow -t_{\beta }^{-1}$	$s_{\alpha }/ s_{\beta } \rightarrow -t_{\beta }^{-1}$	$s_{\alpha }/ s_{\beta } \rightarrow -t_{\beta }^{-1}$
$\xi _H^d$	$s_{\alpha }/ s_{\beta } \rightarrow -t_{\beta }^{-1}$	$c_{\alpha }/ c_{\beta } \rightarrow t_{\beta }$	$s_{\alpha }/ s_{\beta } \rightarrow -t_{\beta }^{-1}$	$c_{\alpha }/ c_{\beta } \rightarrow t_{\beta }$
$\xi _H^l$	$s_{\alpha }/ s_{\beta } \rightarrow -t_{\beta }^{-1}$	$c_{\alpha }/ c_{\beta } \rightarrow t_{\beta }$	$c_{\alpha }/ c_{\beta } \rightarrow t_{\beta }$	$s_{\alpha }/ s_{\beta } \rightarrow -t_{\beta }^{-1}$
$\xi _A^u$	$t_{\beta }^{-1}$	$t_{\beta }^{-1}$	$t_{\beta }^{-1}$	$t_{\beta }^{-1}$
$\xi _A^d$	$-t_{\beta }^{-1}$	$t_{\beta }$	$-t_{\beta }^{-1}$	$t_{\beta }$
$\xi _A^l$	$-t_{\beta }^{-1}$	$t_{\beta }$	$t_{\beta }$	$-t_{\beta }^{-1}$

where the mass parameters $m_{11},m_{22},m_{12}$ should satisfy

38 $$\begin{aligned}&m_{11}^2+\frac{\lambda _1 v^2 \cos ^2\beta }{2}+\frac{\lambda _3 v^2 \sin ^2\beta }{2}\nonumber \\&\quad =\tan \beta \left[ m_{12}^2-(\lambda _4+\lambda _5)\frac{v^2 \sin 2\beta }{4}\right] ,\nonumber \\&m_{22}^2+\frac{\lambda _2 v^2 \sin ^2\beta }{2}+\frac{\lambda _3 v^2 \cos ^2\beta }{2}\nonumber \\&\quad = \frac{1}{\tan \beta } \left[ m_{12}^2-(\lambda _4+\lambda _5)\frac{v^2 \sin 2\beta }{4}\right] . \end{aligned}$$

Later on, we will include these constraints as well when doing our scans.

The coupling of the SM fermions with the Higgses are described by the following lagrangian:

39 $$\begin{aligned}&-\mathcal{L}_{yuk}^{SM} =\sum \limits _{f=u,d,l} \frac{m_f}{v} [\xi ^f_h \overline{f}f h+\xi ^f_H \overline{f}f H-i \xi ^f_A \overline{f}\gamma _5 f A ] \nonumber \\&\quad -\left[ \frac{\sqrt{2}}{v} \overline{u} \left( m_u \xi ^u_A P_L + m_d \xi ^d_A P_R \right) d H^+ \right. \nonumber \\&\qquad \qquad \left. +\frac{\sqrt{2}}{v} m_l \xi _A^l \overline{\nu _L} l_R H^+ + \mathrm {h.c.} \right] , \end{aligned}$$

where the parameters $\xi ^f_{h,H,A}$ depend the couplings of the SM fermions with the two doublets $H_{1,2}$. Motivated by the non-observation of flavor-changing neutral currents (FCNCs) we consider four different sets of $\xi ^f_{h,H,A}$ corresponding to four 2HDM models, i.e. type-I, type-II, lepton-specific and flipped, featuring the absence of tree-level FCNCs [59]. The values of the $\xi$ for these four flavor-conserving types of 2HDMs are listed below in Table 1. On the contrary, we assume generic couplings of the VL fermions to both $H_{1,2}$ doublets^7^:

40 $$\begin{aligned}&-\mathcal{L}_\mathrm{VLL} = y_i^{U_R} \overline{\mathcal{D}_L} \tilde{H}_i U^\prime _R + y^{U_L}_i \overline{U^\prime _L} \tilde{H}_i^\dagger \mathcal{D}_R\nonumber \\&\quad +y^{D_R}_i \overline{\mathcal{D}_L} H_ i D^\prime _R + y^{D_L}_i \overline{D^\prime _L} H_ i^\dagger \mathcal{D}_R \nonumber \\&\quad +M_{\mathcal {D}} \overline{\mathcal{D}_L} \mathcal{D}_R + M_U \overline{U^\prime _L} U^\prime _R\nonumber \\&\quad +M_D \overline{D^\prime _L} D^\prime _R + \mathrm{h.c.}, \end{aligned}$$

where a sum over $i=1,2$ is implied. It is possible to define the Yukawa couplings, $y_h^X$ and $y_H^X$, by the physical CP-even states through the following rotations:

41 $$\begin{aligned}&\begin{pmatrix} y_h^X \\ y_H^X \end{pmatrix} = \begin{pmatrix} c_{\beta } &{} s_{\beta } \\ s_{\beta } &{} -c_{\beta } \end{pmatrix} \begin{pmatrix} y_1^X \\ y_2^X \end{pmatrix}, \nonumber \\&\begin{pmatrix} H_\mathrm{SM} \\ H_\mathrm{NP} \end{pmatrix} = \begin{pmatrix} c_{\beta } &{} s_{\beta } \\ s_{\beta } &{} -c_{\beta } \end{pmatrix} \begin{pmatrix} H_1 \\ H_2 \end{pmatrix}, \end{aligned}$$

where we used the superscript $X = U_{L/R}$ or $D_{L/R}$. As we are working in the alignment limit, $H_\mathrm{SM}$ becomes the SM Higgs double, while $$H_\mathrm{NP} = \begin{pmatrix} H^+ \\ (H-iA)/\sqrt{2} \end{pmatrix}$$. Since we are coupling the VL fermions to both doublets, the value of $t_{\beta }$ or the chosen type of 2HDM will be irrelevant for the VLF coupling to the scalars. On the contrary, the Yukawa couplings of the SM fermions are dictated exactly by the choices of $t_{\beta }$ and of the 2HDM type.

A DM candidate is again straightforwardly introduced by considering a lagrangian of the form (40) with $U \equiv N$ and $D \equiv E$. Our analysis will substantially follow the same lines as in the case of VLL extensions of the SM Higgs sector. Before determining the DM observables and comparing them with experimental constraints, we will reformulate, in the next subsections, for the case of the 2HDM, the constraints from the SM Higgs signal strength and from EWPT. We will also consider an additional set of constraints, which influence the size of the new Yukawa couplings, from the UV behavior of the theory.

### Higgs signal strengths

Having imposed the alignment limit, the extended Higgs sector does not influence the decay branching fractions of the 125 GeV SM-like Higgs. The only possible source of deviation from the SM expectation is represented by the VLLs, which can affect the $h\rightarrow \gamma \gamma$ signal strength, $\mu _{\gamma \gamma }$. The corresponding contribution substantially coincides with the one determined in the one Higgs doublet scenario, namely Eq. (8). Assuming the presence of only one family of VLLs, the simplest solution for having an experimentally viable scenario is to set to zero one of the $y_h^{E_{L,R}}$ couplings. Unless stated otherwise, we will assume, in the analysis below, that $y_h^{E_R}=0$.

### EWPT constraints

In a 2HDM$+$VLL framework new contributions, with respect to the SM, to the *S* and *T* parameters originate from both the fermionic and the scalar sector. As regards the former, these contributions depend, as for the case of one Higgs doublet, on the masses of the new fermions and their couplings $y_h^{N_{L,R},E_{L,R}}$ with the SM-like Higgs, while the couplings with the other Higgs states are unconstrained. The contributions from the scalar sector are instead related to the masses of the new Higgs states. Also in this case it is possible to forbid deviations from the SM expectations of the *T* parameter by imposing a custodial symmetry. In the alignment limit this is realized by setting $m_H \simeq m_{H^\pm }$ or $m_A \simeq m_{H^\pm }$ [46, 69] and considering only constraints from the *S* parameter. As already pointed out and further clarified below, this choice would imply excessive limitations to DM phenomenology. For this reason we will not impose a custodial symmetry, neither to the fermionic nor to the scalar sector, but rather freely vary the corresponding parameters and require in turn that the *S*, *T* parameters do not deviate by more than $3\sigma$ from their best fit values.

**Fig. 5:**
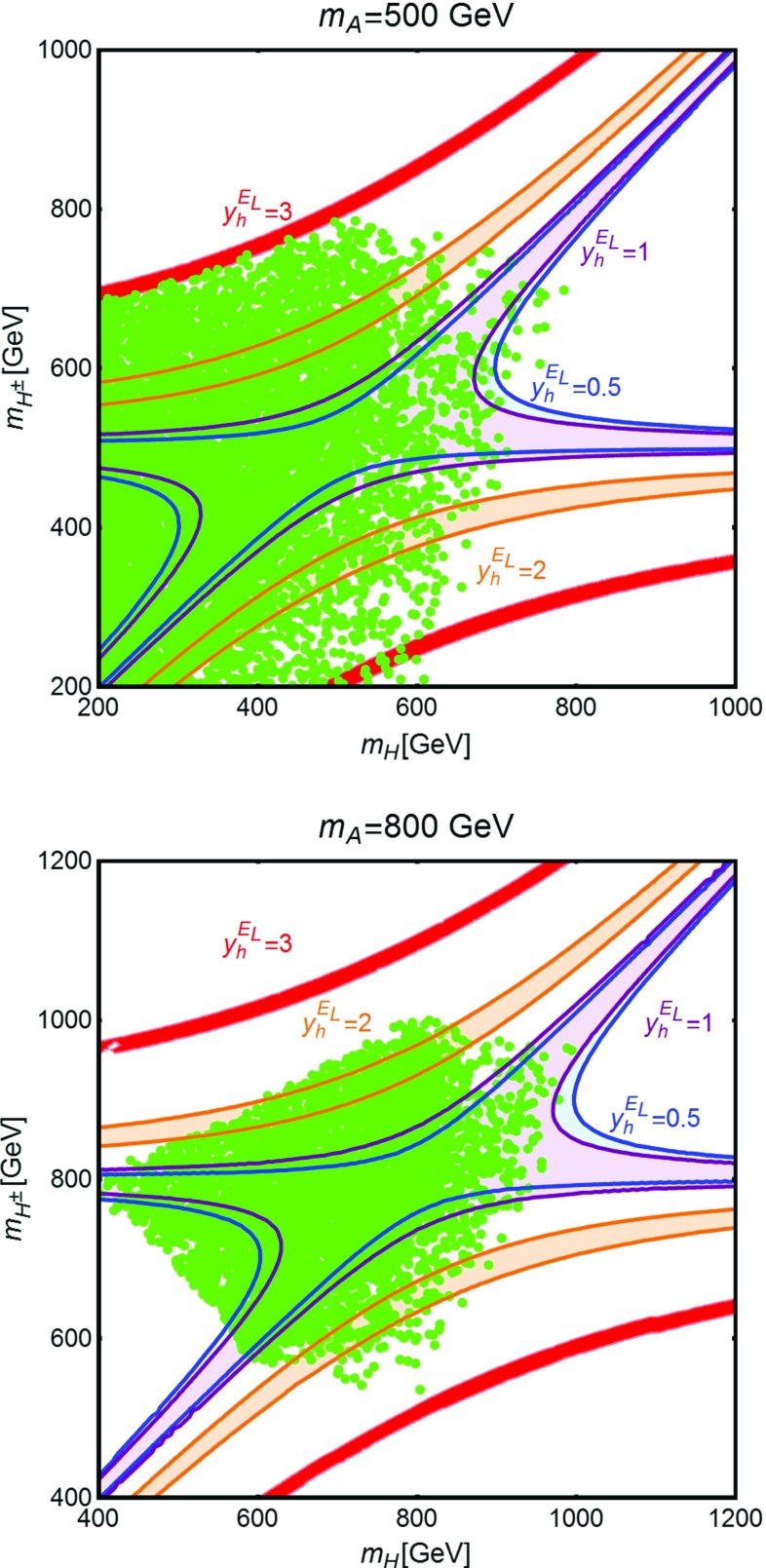
Impact of EWPT constraints in the bidimensional plane $(m_H,m_{H^{\pm }})$ for two fixed assignations of $m_A$, i.e. 500 and 800 GeV. The *blue*, *purple*, *orange* and *red* regions represent the allowed parameter space for, respectively, $y_h^{E_L}=0.5,1,2,3$. The *green points* represent the configurations allowed by the constraints reported in Eqs. (34) and (35)

For illustrative purposes we have reported in Fig. 5 the regions allowed by EWPT for some definite assignation of model parameters. More specifically we have fixed the values of the DM candidate $M_{N_1}$ and of the lightest charged new fermion $m_{E_1}$, as well as the Yukawa coupling $y_h^{N_L}$, to, respectively, 120 GeV, 250 GeV and 0.01 (this very low value is motivated by constraints from DM DD), while we have varied the parameter $y_h^{E_L}$, since it will be relevant for the DM relic density as well as for LHC detection prospects. Regarding the scalar sector we have fixed $m_A=500$ GeV (left panel) and $m_A=800$ GeV (right panel) and varied the mass of the CP-even Higgs state *H* and of the charged one $H^{\pm }$. For $y_h^{E_L} \le 1$ the effect of the fermionic sector on the EWPT is subdominant such that the allowed regions substantially corresponds to the one allowed in the case of no VLLs present in the theory. On the contrary, once the value of $y_h^{E_L}$ is increased, a cancellation between the contributions from the fermionic and scalar sectors is needed in order to comply with experimental constraints. As a consequence, the allowed regions of the parameter space are reduced to rather narrow bands. We also notice that, in this last case, the constraints from EWPT disfavor mass degenerate $H,A,H^{\pm }$. We recall that, on the other hand, the variation of the masses of the Higgs states is constrained by perturbativity and unitarity limits, Eqs. (34) and (35). We have then reported on Fig. 5 the regions allowed by the latter constraints, determined by varying the input parameters of Eq. (29) over the same ranges considered in [66] (contrary to this reference we have nevertheless assumed alignment limit). As we can see, values of $y_h^{E_L}$ above 3 are excluded for $m_A=500\,\text{ GeV }$ while for $m_A=800\,\text{ GeV }$ we get the even stronger constraint $y_h^{E_L} \lesssim 2$.

### Constraints from RGE evolution

The extension of the Higgs sector with VLFs suffers also constraints from theoretical consistency. Indeed, the presence of new fermions affects the RGE evolution of the parameters of the 2HDM, in particular the gauge couplings and the quartic couplings of the scalar potential [70], making it difficult for the new states to induce sizable collider signals, like diphoton events [71–78] (see also below).

As regards the gauge couplings, their $\beta$ functions receive a positive contribution depending on the number of families of vector-like fermions and on their quantum numbers under the SM model gauge group. In case that these contributions are too high the gauge couplings can be lead to a Landau pole at even moderate/low energy scales. However, in the case considered in this work, i.e. one family of vector-like leptons, we have only a small contribution to the $\beta$ functions of the couplings $g_1$ and $g_2$ which does not affect in a dangerous way their evolution with energy.

Very different is, instead, the case of the quartic couplings. As already seen in the case of a SM-like Higgs sector, the radiative corrections associated to the VLLs depend on their Yukawa couplings. The $\beta$ functions are given by

42 $$\begin{aligned}&\beta _{\lambda _1}=\beta _{\lambda _1,\mathrm 2HDM}+\frac{1}{8 \pi ^2}\left( \lambda _1 \sum _L |y_1^L|^2 -\sum _L |y_1^L|^4\right) , \end{aligned}$$

43 $$\begin{aligned}&\beta _{\lambda _2}=\beta _{\lambda _2,\mathrm 2HDM}+\frac{1}{8 \pi ^2}\left( \lambda _2 \sum _L |y_2^L|^2 - \sum _L |y_2^L|^4\right) , \end{aligned}$$

44 $$\begin{aligned}&\beta _{\lambda _3}=\beta _{\lambda _3, \mathrm 2HDM}+\frac{1}{16 \pi ^2} \biggl (\lambda _3 \sum _L (|y_1^L|^2+|y_2^L|^2) \nonumber \\&\quad -2 y_1^{E_L} y_2^{E_L} y_1^{N_L}y_2^{N_L}+(|y_1^{N_L}|^2+|y_1^{E_L}|^2)(|y_2^{N_L}|^2+|y_2^{E_L}|^2)\nonumber \\&\quad -2 y_1^{E_R} y_2^{E_R} y_1^{N_R}y_2^{N_R}+(|y_1^{N_R}|^2+|y_1^{E_R}|^2)(|y_2^{N_R}|^2+|y_2^{E_R}|^2)\biggr ), \end{aligned}$$

45 $$\begin{aligned}&\beta _{\lambda _4}=\beta _{\lambda _4, \mathrm 2HDM}+\frac{1}{16 \pi ^2} \biggl (\lambda _4 \sum _L (|y_1^L|^2+|y_2^L|^2) \nonumber \\&\quad -2 y_1^{E_L} y_2^{E_L} y_1^{N_L}y_2^{N_L}+(|y_1^{N_L}|^2-|y_1^{E_L}|^2)(|y_2^{N_L}|^2-|y_2^{E_L}|^2)\nonumber \\&\quad +2 y_1^{E_R} y_2^{E_R} y_1^{N_R}y_2^{N_R}+(|y_1^{N_R}|^2-|y_1^{E_R}|^2)(|y_2^{N_R}|^2-|y_2^{E_R}|^2)\biggr ), \end{aligned}$$

46 $$\begin{aligned} \beta _{\lambda _5}&=\beta _{\lambda _5,\mathrm 2HDM}+\frac{1}{16 \pi ^2}\nonumber \\&\quad \bigg ( \lambda _4 \sum _L (|y_1^L|^2+|y_2^L|^2) -2 \sum _L |y_1^L|^2 |y_2^L|^2\bigg ), \end{aligned}$$

where $\beta _{\lambda _i,\mathrm 2HDM}$ are the contributions to the $\beta$ function originating only from the quartic couplings themselves and the Yukawa couplings of the SM fermions. We refer to [59] for their explicit expressions.

To simplify the notation we have expressed, in Eqs. (42)–(46),^8^ the Yukawa couplings in the $(H_1,H_2)$ basis.

As evident, the quartic couplings receive large radiative corrections scaling either with the second or the fourth power of the Yukawa couplings. As a consequence, vacuum stability and/or perturbativity and unitarity might be spoiled at some given energy scale unless additional degrees of freedom are introduced in the theory.

A quantitative analysis would require the solution of Eqs. (42)–(46) coupled with RGE for the gauge and Yukawa couplings as function of the masses of the Higgs eigenstates and the parameters *M* and $t_\beta$, which determine the initial conditions for $\lambda _{1,5}$, and verify conditions 34 and 35 as function of the energy scale. A good qualitative understanding can be nevertheless achieved by noticing that for sizable Yukawa couplings the $\beta$ functions (42)–(46) are dominated by the negative contributions scaling with the fourth power of the Yukawas themselves (their $\beta$ function are positive, scaling qualitatively as $\beta _y \propto y^3$). As a consequence one can focus, among 34 and 35, on the conditions $\lambda _{1,2}>0$. In analogous fashion to the case of the SM Higgs sector, discussed in the previous section, we will just require low energy viability of the model. In other words, a given set of model parameters will be regarded as (at least phenomenologically) viable if the scale at which the couplings $\lambda _1,\lambda _2$ become negative is considerably above few TeVs, i.e. far enough from the energy scales probed by collider processes. Additional degrees of freedom at high energy scale might, at this point, improve the behavior of the theory. The study of explicit scenarios is anyway not in the purposes of this study.

According to the discussion above, in a phenomenologically viable setup, the quartic couplings $\lambda _1$ and $\lambda _2$ should not vary too fast with the energy. As proposed in [79], a good approximate condition consists in imposing $|\beta _{\lambda _{1,2}}/\lambda _{1,2}| < 1$, with $\lambda _{1,5}$ computed according to Eq. (29) and the Yukawa couplings set to their input value at the EW scale. In case this condition is not fulfilled, the functions $\beta _{\lambda _{1,2}}$ would vary too fast with the energy so that the theory would manifest a pathological behavior already in proximity of the energy threshold corresponding to the masses of the VLLs.^9^

As already pointed out, the requirements of a reliable behavior of the theory under RG evolution mostly affect possible predictions of LHC signals. As will be reviewed in greater detail in the next subsections, one of the most characteristic signatures induced by the VLLs are enhanced diphoton production rates from decays of resonantly produced *H* / *A* states. This happens because their effective couplings with photons are increased by triangle loops of electrically charged VLLs such that, once their masses are fixed, the corresponding rate depends on the size of the Yukawa couplings. The constraints from RGE can be used to put an upper limit on the size of the Yukawa couplings which imply, in turn, an upper limit on the diphoton production cross sections which are expected to be observed.

**Fig. 6:**
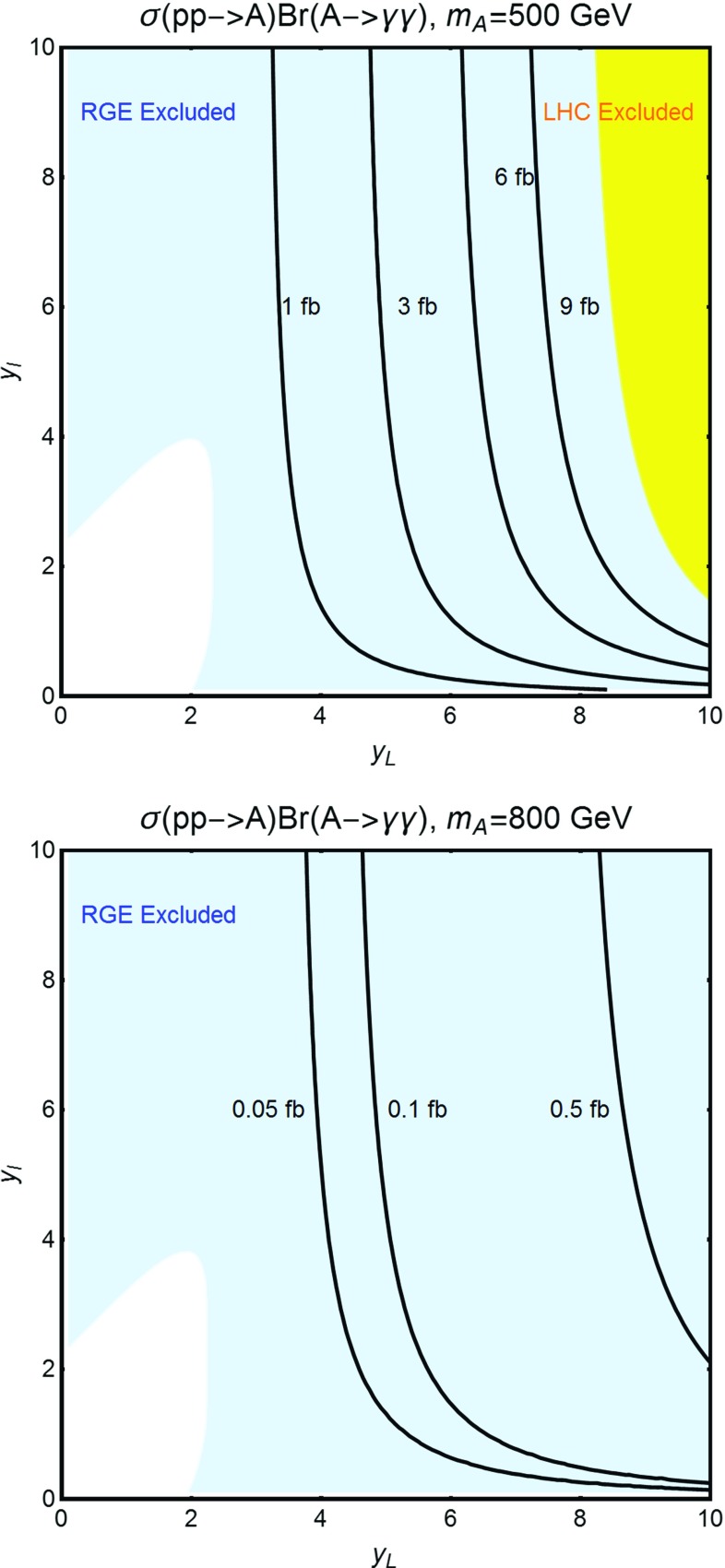
Contours of the process $pp \rightarrow A \rightarrow \gamma \gamma$ for the two values $m_A=500\,\text{ GeV }$ (*upper panel*) and $m_A=800\,\text{ GeV }$ (*lower panel*), as function of the parameters $y_{l,L}$ (see main text). In both plots we have considered type-I 2HDM with $\tan \beta =1$. The *yellow region in the left panel* is excluded by present LHC searches. In the *region at the left* of the 1 fb (*upper panel*) and 0.05 fb (*lower panel*) contours, the production cross section varies in a negligible way with $y_{l,L}$ and basically coincides with the prediction of the 2HDM without VLLs. The *blue region* corresponds to theoretically inconsistent, because of RGE effects, values of the Yukawa parameters

As illustration we have thus reported in Fig. 6 the isocontours of $\sigma ( pp \rightarrow A) Br(A \rightarrow \gamma \gamma )$ as function of $y_l=y_h^{E_L}$ and $y_L=y_H^{E_L}=-y_H^{E_R}=-y_H^{N_L}=y_H^{N_R}$ (see below for clarification), for two values of $m_A$, namely 500 and 800 GeV. As further assumption we have set $m_{E_1}=m_A/2$ in order to maximize the effective coupling between *A* and the photons.^10^

Clearly, in order to obtain sensitive deviations from the prediction of a 2HDM without VLLs, which is approximately 1 and 0.05 fb for the two examples considered, rather high values of the new Yukawas are needed,^11^ which would induce too large radiative corrections to the quartic couplings of the scalar potential. In theoretically consistent realizations, the VLLs have negligible effects on the diphoton production cross section.

**Fig. 7:**
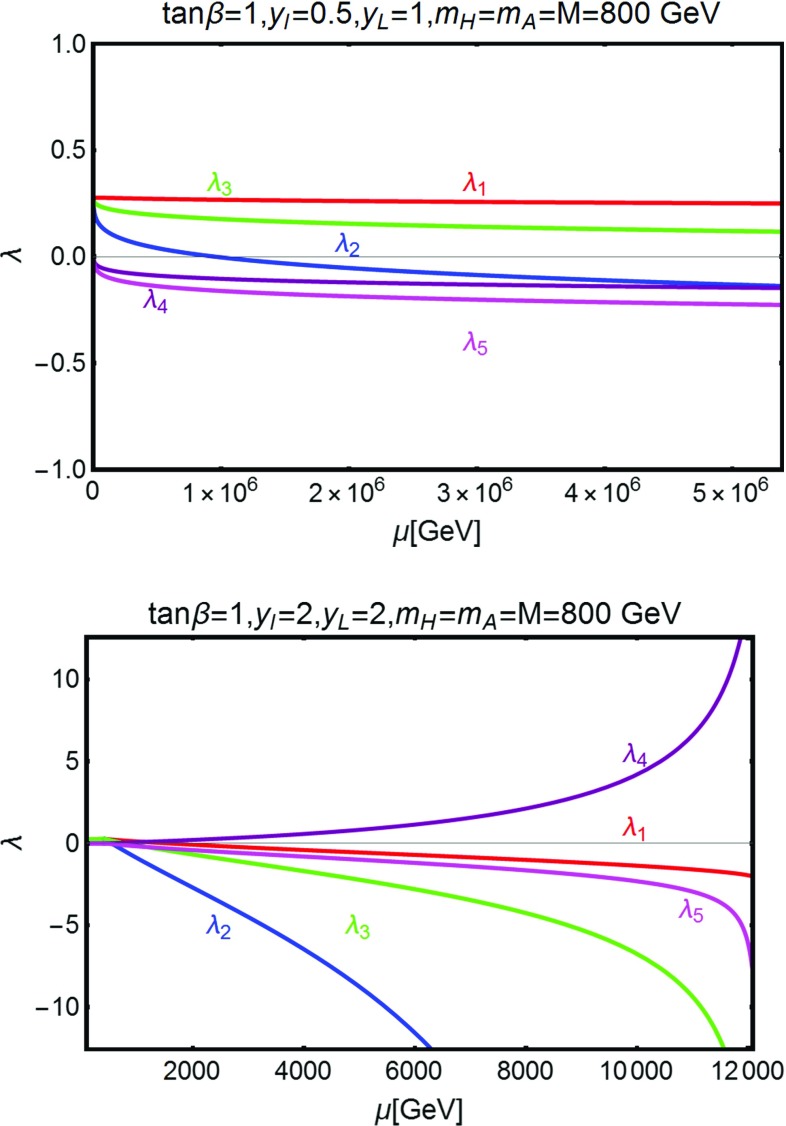
Two examples of resolution of the RGE equations. The corresponding assignations of the relevant model parameters are reported on *top of the panels*. In the *upper panel* the initial values of the Yukawa couplings are sufficiently small such that the conditions (34) and (35) are satisfied up to energy scales of the order of $10^6\,\text{ GeV }$. In the *lower panel* the assignation of the Yukawas causes, instead, the couplings $\lambda _{1,2}$ to become negative already at the energy threshold of the VLLs

We have checked the validity of the criteria $|\beta _{\lambda _{1,2}}/\lambda _{1,2}|\le 1$ by explicitly solving the RGE for some benchmark models. We have reported two sample solutions in Fig. 7. Here we have considered the same assignation of the model parameters as in the lower panel of Fig. 6, and we have chosen two assignations of the input values of the Yukawa parameters $y_{l,L}$. In the upper panel we have considered the set $(y_l,y_L)=(0.5,1)$, lying in the white region of the lower panel of Fig. 6. Evidently, the couplings $\lambda _{1,2}$ remain positive up to an energy scale $\mu$ of the order of $10^6\,\text{ GeV }$, sufficiently high for the model point to be viable at least as a phenomenological description.^12^ On the contrary, by considering a parameter assignation lying in the blue region, RGEs drive the couplings $\lambda _{1,2}$ to negative values already at the energy threshold of the charged VLLs, of the order of 400 GeV for the case considered.

### DM phenomenology

The coupling of the DM to an additional Higgs doublet has a twofold impact on dark matter phenomenology. First of all, the extra neutral Higgs states constitute additional s-channel mediators for DM annihilations and, only for the case of *H*, t-channel mediators for scattering processes relevant for direct detection. In addition, in the high DM mass regime, they may represent new final states for DM annihilation processes.

The coupling of the DM with the non-SM-like Higgs states can be expressed, in the mass basis, in terms of the Yukawa couplings $y_{h,H}^X$ and of the mixing angles $\theta _X^{L,R}$:

47 $$\begin{aligned}&y_{H N_1 N_1}= \frac{\cos \theta _N^L \sin \theta _N^R y_H^{N_L}+\cos \theta _N^R \sin \theta _N^L y_H^{N_R}}{\sqrt{2}}, \nonumber \\&y_{A N_1 N_1}= i\frac{\cos \theta _N^L \sin \theta _N^R y_H^{N_L}-\cos \theta _N^R \sin \theta _N^L y_H^{N_R}}{\sqrt{2}}, \nonumber \\&y_{H^+ N_1 E_1}=\cos \theta _N^L \sin \theta _E^R y_H^{E_L}+\sin \theta _N^L \cos \theta _E^R y_H^{E_R}\nonumber \\&\quad -\cos \theta _N^R \sin \theta _E^L y_H^{N_R}-\cos \theta _N^L \sin \theta _E^R y_H^{N_L}. \end{aligned}$$

The analysis of the DM phenomenology is structured in an analogous way as the one performed in the previous section. We will compute the DM annihilation cross section and verify for which assignations of the parameters of the model the thermally favored value, $\sim 3 \times 10^{-26}\,{\text{ cm }}^3\, {\text{ s }}^{-1}$, is achieved without conflicting with bounds from DM direct detection. Given the dependence of the coupling between the DM and the neutral Higgs states on the mixing angles $\theta _N^{L,R}$ the DM scattering cross section is still dominated by the *Z* exchange processes so that the new couplings from Eq. (47) mostly impact the determination of the DM relic density.

As regards the DM relic density, we distinguish several possibilities:

- $m_{N_1} \le m_{X}/2,X=A,H,H^{\pm }$ and sizable mass splitting between the DM and the other vector-like fermions. In this case the situation is very similar to the case of SM$+$VLLs. The most relevant DM annihilation channels are again into fermion and gauge boson pairs. Recalling that, in the alignment limit, there is no tree-level coupling between the *H*, *A* states and the *W*, *Z* bosons, the only annihilation processes substantially influenced by the presence of the additional Higgs bosons are the ones into SM fermions. In particular, s-channel exchange of the CP-odd Higgs gives rise to a new s-wave contribution so that the DM annihilation cross section can be schematically written
  48 $$\begin{aligned}&\langle \sigma v \rangle _{ff}=\frac{m_{N_1}^2}{8 \pi } \frac{m_t^2}{v^2}|\xi _{A}^t|^2 \frac{1}{((4 m_{N_1}^2-m_A^2)^2+m_A^2 \Gamma _A^2)}|y_{A N_1 N_1}|^2 \nonumber \\&\quad + \frac{g^2 m_{N_1}^2}{\pi ((4 m_{N_1}^2-m_Z^2)^2+m_Z^2 \Gamma _Z^2)}\nonumber \\&\quad \times \left[ \sum n_c^f (|V_f|^2+|A_f|^2) |y_{V,Z N_1 N_1}|^2\right. \nonumber \\&\quad \left. +\frac{3 m_t^2}{2 m_{N_1}^2}(|V_t|^2+|A_t|^2)|y_{A,Z N_1 N_1}|^2\right] . \end{aligned}$$
  Evidently, the annihilation cross section depends, through the factor $\xi$, on $\tan \beta$ and, in turn, on the realization of the couplings of the two Higgs doublets to SM fermions. Given the dependence on the mass of the final state fermions, *A*-exchange diagrams give a sizable contribution mostly to the $\bar{t} t$ final state, when kinematically open (an exception being a type-II/flipped 2HDM for $\tan \beta \gtrsim 45$, when a sizable contribution comes also from $\bar{b} b$).

**Fig. 8:**
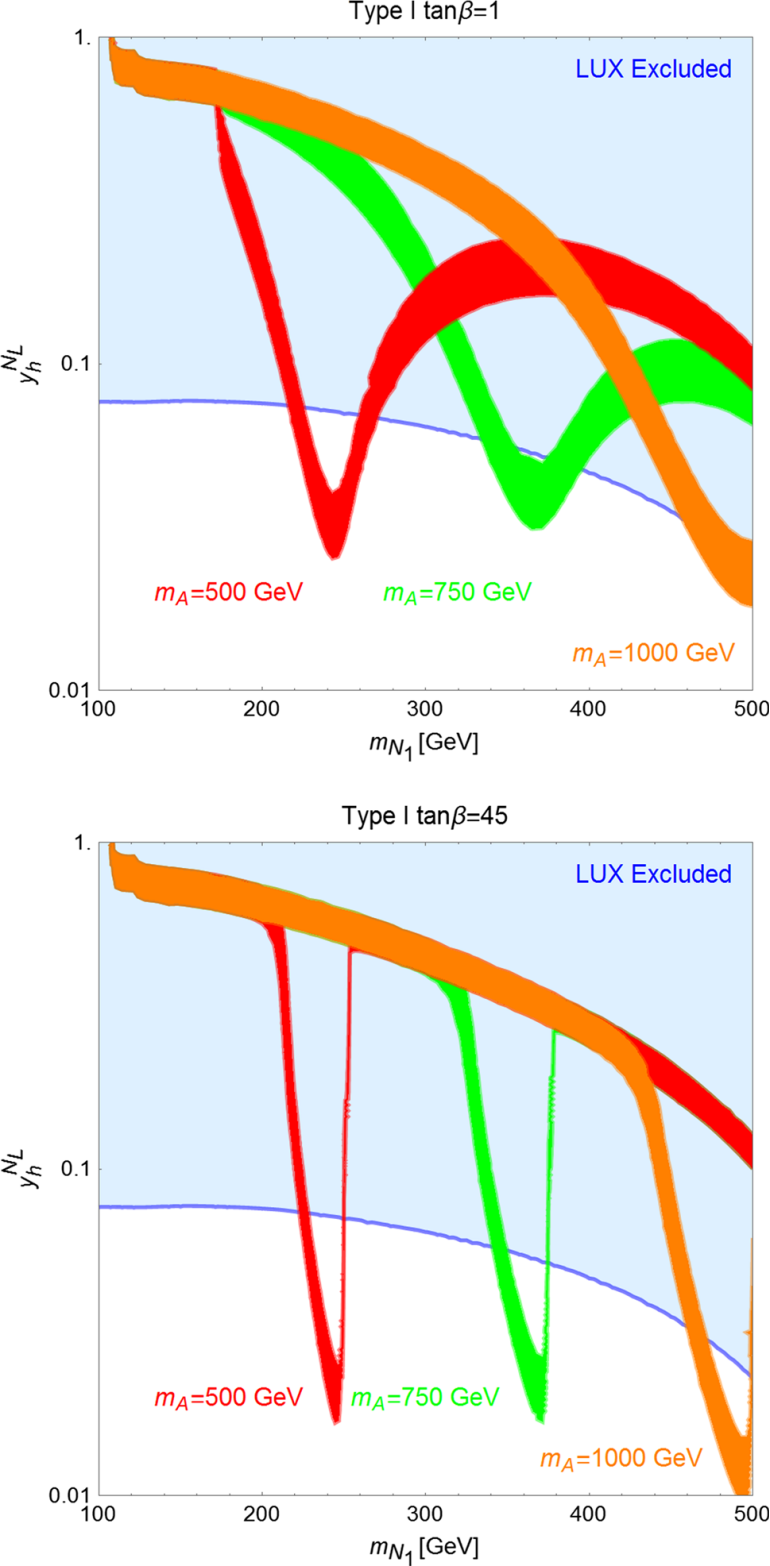
Isocontours of the correct DM relic density in the bidimensional plane $(m_{N_1},y_h^{N_L})$ for two values of $\tan \beta$, (*upper panel*) 1 and (*lower panel*) 45, and for the following assignations of the other parameters of the fermion sector: $y_h^{N_R}=y_h^{E_R}=0$, $y_h^{E_L}=0.5$, $y_H^{N_L}=-y_H^{N_R}=-y_H^{E_L}=y_H^{E_R}=1$. We have finally set $M=m_H=m_A=m_{H^{\pm }}$ and considered the three values of 500 GeV, 750 GeV and 1 TeV

As already pointed out, the strong DD limits, mostly originating from t-channel *Z* exchange, impose the requirement that the DM is essentially a pure *SU*(2) singlet with, as well, a tiny hypercharged component. This implies also a suppression of the couplings of the DM to the neutral Higgs states, such that the DM is typically overproduced in the parameter regions compatible with DD constraints. It is nevertheless possible to achieve the correct relic density by profiting of the resonant enhancement of the DM annihilation cross section when the condition $m_{N_1}\simeq \frac{m_{H,A}}{2}$ is met. Notice that in this case the DM annihilation cross section is also sensitive to the total width of the *H* / *A* state and thus sensitive to the value of $\tan \beta$. An illustration of the DM constraints in the $m_{N_1} \le \frac{m_{A,H}}{2}$ regime is provided in Fig. 8. Here we have compared, for two values of $\tan \beta$ (for definiteness we have considered type-I 2HDM), the isocontours of the correct DM relic density, for three assignations of $m_A=m_H=m_{H^{\pm }}$ (for all the three cases we set $M_E=M_L$ so that the lightest charged VLL, $E_1$, has a mass of 500 GeV), and the DD exclusion limit, as set by LUX. As already anticipated the only viable regions are the ones corresponding to the s-channel poles. We also notice that the shapes of the relic density contours are influenced by the large (narrow) widths of the resonances occurring for small (high) $\tan \beta$.

**Fig. 9:**
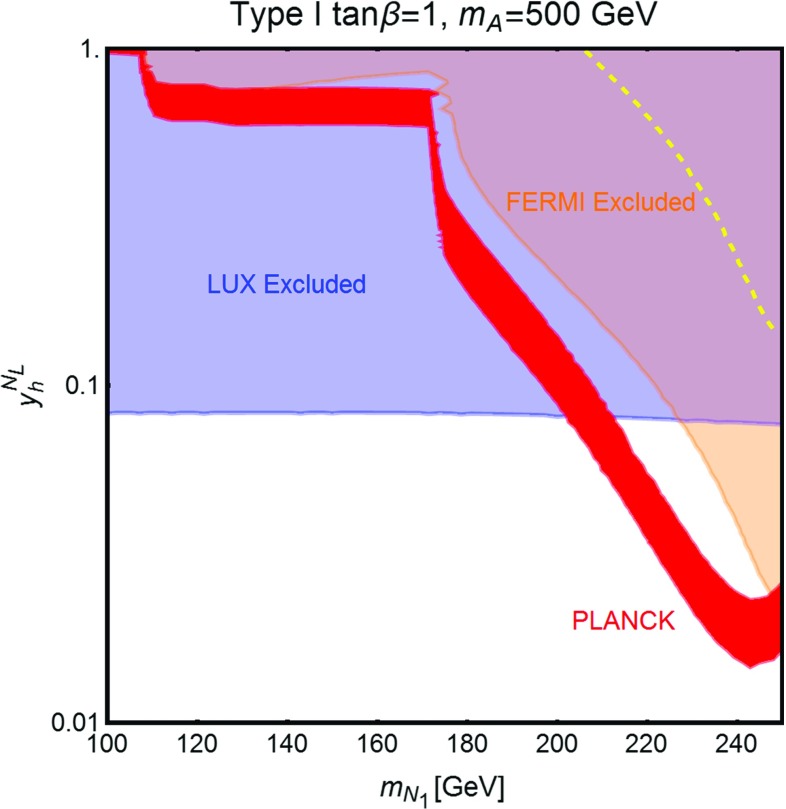
Comparison between the different DM constraints from one of the benchmarks considered in Fig. 8. In addition to the already considered constraints from relic density and DD the figure reports (in *orange*) the excluded region by searches of gamma-rays in DSph [81] as well as the limit (*yellow dashed line*) from gamma-ray lines [82]

Concerning indirect detection, possible signals might originate with residual annihilation processes, at present times, into $\bar{f} f$ (mostly $\bar{b} b$ and $\bar{t} t$ when kinematically accessible), $W^+ W^-$, *ZZ* and *Zh*, since their corresponding annihilation cross section have unsuppressed s-wave (i.e. velocity independent) contributions. These annihilation processes can be probed by searches of gamma-rays produced by interactions of the primary products of DM annihilation. The most stringent constraints come from searches in Dwarf Spheroidal galaxies (DSph) [81]. These kinds of constraints can probe thermally favored values of the DM annihilation cross section only for DM masses below 100 GeV. As evidenced in Fig. 9 they are then considerably less competitive, with the exception of the resonance region, than the ones from DD. An additional indirect signal might be represented by gamma-ray lines produced in the annihilation process $N_1 N_1 \rightarrow \gamma \gamma$ originate with a one-loop-induced effective vertex between the pseudoscalar Higgs state A and two photons [6, 83]. In our setup this annihilation channel is, however, rather suppressed so that it is not capable to probe the thermal DM region (see dashed yellow line in Fig. 9).

- $m_{N_1} < m_{X}/2,X=A,H,H^{\pm }$ and DM close in mass with at least the lightest charged VLL. In this case the DM relic density is not only accounted for by pair annihilation of the DM particle $N_1$ but also by coannihilation processes of the type $N_i E_{j} \rightarrow \bar{f} f', W^{\pm }h, W^{\pm } Z,\,i,j=1,2$ 13 (in most of our computations we have assumed $M_E=M_L$ and, then, the charged eigenstates are very close in mass) which occur through s-channel exchange of the $W^{\pm }$ and the $H^{\pm }$ or t-channel exchange of the VLLs themselves. These kinds of process can easily be dominant, providing a low enough mass splitting, with respect to $N_1 N_1$ annihilation since their corresponding annihilation rates depend on the couplings $y_h^{E_L},y_H^{E_{L,R}}$ which are not subject to the strong constraints from DD. We also notice that coannihilations would be relevant in the case that a custodial symmetry is imposed in the VLF sector.

**Fig. 10:**
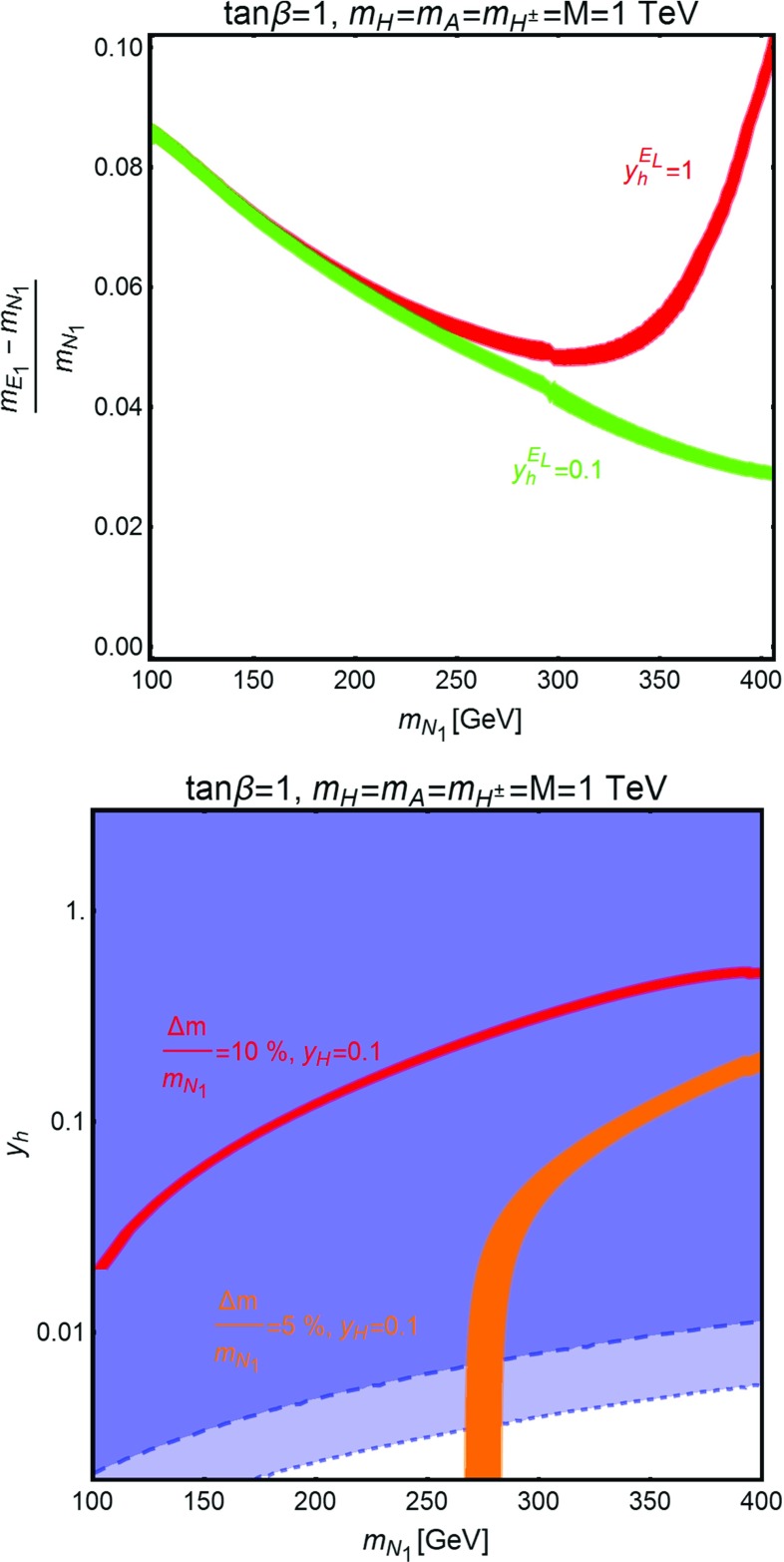
*Top panel* isocontours of the correct DM relic density in the bidimensional plane $(m_{N_1},\frac{m_{E_1}-m_{N_1}}{m_{N_1}})$ for $\tan \beta =1$, $y_h^{N_R}=y_h^{E_R}=0$, $y_h^{N_L}=5\times 10^{-3}$, and two assignations of $y_h^{E_L}=y_H^{N_L}=-y_H^{N_R}=-y_H^{E_L}=y_H^{E_R}$, i.e. 0.1 and 1. Notice that we have set $M=m_H=m_A=m_{H^{\pm }}=1\,\text{ TeV }$. *Bottom panel* isocontours of the correct relic density, assuming $y_h^{N_L}=y_h^{E_L}$, for two values of $\frac{m_{E_1}-m_{N_1}}{m_{N_1}}$, namely 5 and $10\%$, and the corresponding excluded region by LUX, in, respectively, *blue* and *dark blue*

The DM phenomenology in the presence of coannihilations is illustrated in Fig. 10. We have reported, in the top panel, the isocontours of the correct DM relic density in the bidimensional plane $(m_{N_1},\frac{m_{E_1}-m_{N_1}}{m_{N_1}})$. For simplicity we have assumed $y_h^{E_L}=y_H^{N_L}=-y_H^{N_R}=-y_H^{E_L}=y_H^{E_R}$ and considered two numerical values, i.e. 0.1 and 1. The remaining non-zero coupling, $y_h^{N_L}$, has been set to $10^{-3}$ in order to evade constraints from DD. The masses of the new Higgs states have been finally set to the value of 1 TeV in order to avoid effects on the relic density for DM masses of few hundreds GeV. As evidenced in the figure the correct relic density is achieved, through coannihilation processes, provided that the relative mass splitting between the DM and the lightest charged state is between approximately 2 and $10\%$. We emphasize that we have chosen a much lower value of $y_h^{N_L}$ with respect to the limits shown, for example in Fig. 8. This is because the almost full degeneracy between $m_{N_1}$ and $m_{E_1}$ implies $M_N \simeq M_L$, in turn implying enhancement of the angles $\theta _N^{L,R}$, which set the size of the coupling of the DM with the *Z*, mostly responsible of the SI cross section. This last feature is well evidenced in the bottom panel of Fig. 10 where we have assumed the $y_h^{N_L}=y_h^{E_L}$ limit to easily compare relic density and direct detection. As is evident, the latter is responsible of very strong constraints, reaching almost $y_h^{N_L} \sim 10^{-3}$ and almost excluding the regions at viable DM relic density.

In the case that the DM relic density is mostly accounted by coannihilation processes we do not expect ID signals since the rate of this kind of processes is (Boltzmann) suppressed at present times.

- $m_{N_1} > m_{X}/2,X=A,H,H^{\pm }$ (no coannihilations): in this case the situation is very different from the case of the SM Higgs sector. Indeed, as the DM mass increases, new annihilation channels become progressively open. We have, first of all, when $m_{N_1} > m_{X}/2,X=A,H,H^{\pm }$, the opening of annihilation channels of the type *VX* where $V=Z,W^{\pm },X= A,H,H^{\pm }$. By further increasing the DM mass, annihilation channels into pairs of Higgs states are finally reached. Among these new channels the most efficient turn out to be the ones into $W^{\pm } H^{\mp }$ and into $H^{\pm } H^{\mp }$. Indeed these processes can occur through t-channel exchange of the lightest charged state $E_1$ and the corresponding rates depend on the coupling $y_{H^+ N_1 E_1}$, which depends on parameters not involved in direct detection processes and which might be of sizable magnitude even for a SM singlet DM, provided that the charged state $E_1$ has a sizable *SU*(2) component. The potentially rich phenomenology offered by the annihilations into Higgs–gauge bosons and Higgs boson pairs is the reason why we have not strictly imposed a custodial symmetry in the scalar sector since it would have imposed a too rigid structure to the mass spectrum.

**Fig. 11:**
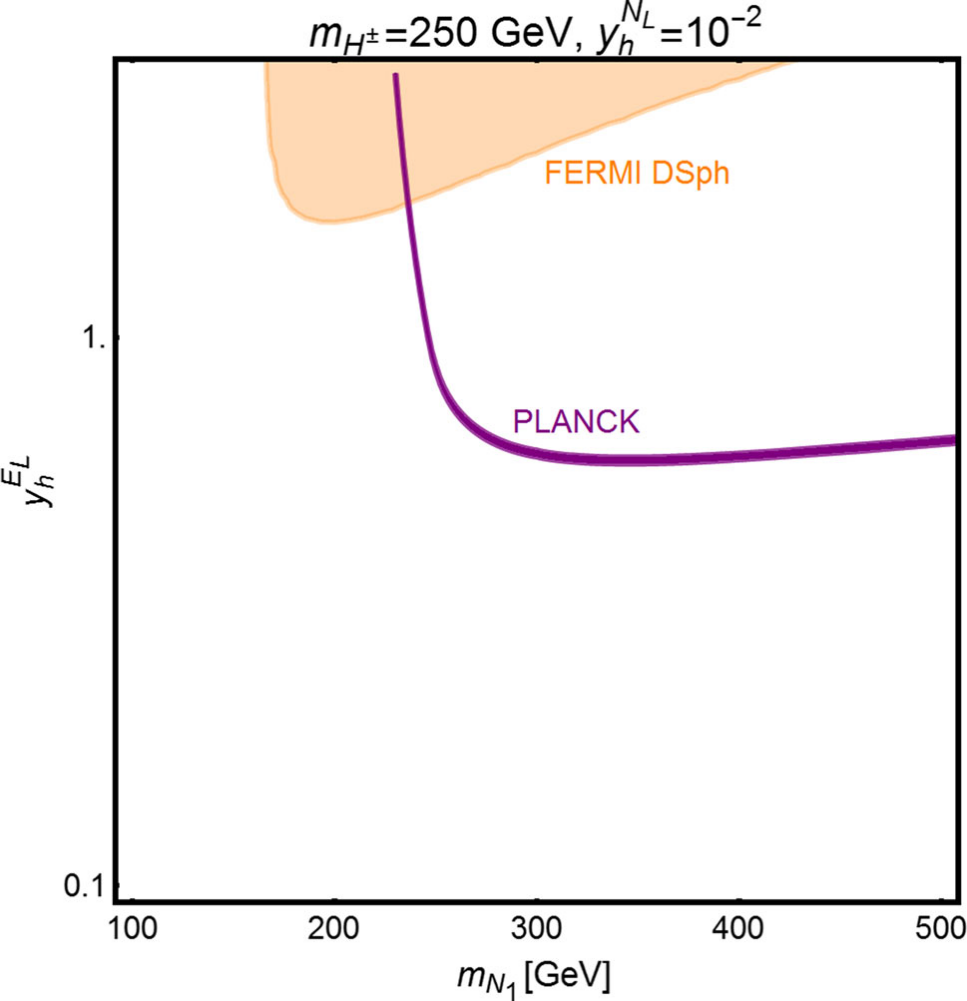
Isocontour (*purple line*) in the bidimensional plane $m_{N_1},y_h^{E_L}$ of the correct DM relic density for $m_{H^{\pm }}=250\, \text{ GeV }$. The *orange region* is excluded by searches of gamma-ray signals in DSph. The coupling $y_h^{N_L}$ has been set to $10^{-2}$ to evade constraints from DM direct detection

Concerning possible indirect detection constraints, these rely on gamma-ray signals originating with cascade decays of the $H^{\pm },W^{\pm }$ [84]. Even though these annihilations are sizable at present times the corresponding cross section has nevertheless a non-negligible velocity dependent component. Consequently, annihilations at present times have a smaller rate than at thermal freeze-out and then ID constraints have a marginal relevance in the region of parameter spaces corresponding to viable DM relic density. This is shown, in one example, in Fig. 11.

In order to explore the multi-dimensional parameter space we have then employed a scan of the following parameters:

49 $$\begin{aligned}&y_h^{N_{L,R}},y_H^{N_{L,R}} \in [10^{-3},1], \nonumber \\&y_h^{E_{L}}, y_H^{E_{L,R}} \in [5 \times 10^{-3},3], \nonumber \\&M_N \in \left[ 100\,\text{ GeV }, 1\,\text{ TeV }\right] , \nonumber \\&M_E=M_L \in \left[ 100\,\text{ GeV }, 1\,\text{ TeV }\right] , \nonumber \\&\tan \beta \in \left[ 1,50\right] ,\nonumber \\&m_A \in \left[ 250\,\text{ GeV },1\,\text{ TeV }\right] ,\nonumber \\&m_H \in \left[ m_h, 1.5\,\text{ TeV }\right] ,\nonumber \\&m_{H^{\pm }} \in \left[ m_W,1.5\,\text{ TeV }\right] , \nonumber \\&|M| \in \left[ 0, 1.5 \,\text{ TeV }\right] , \end{aligned}$$

and we required that the model points pass the constraints from EWPT, from perturbativity and unitarity of the scalar quartic couplings, Eqs. (34) and (35), and from satisfying the requirement of stability under RGEs, $|\beta _{\lambda _{1,2}}/\lambda _{1,2}| <1$. We have finally required that the correct DM relic density is achieved. Similarly to the case discussed in the previous section, we have disregarded the possibility of coannihilations between the DM and other VLLs by further imposing a minimal mass difference between these states. We have repeated this scan for the different 2HDM realizations reported in Table 1. Although the DM results are mostly insensitive to the type of couplings of the Higgs states with SM fermions the prospects for LHC searches, discussed in the next subsection, will be different in the various cases.

**Fig. 12:**
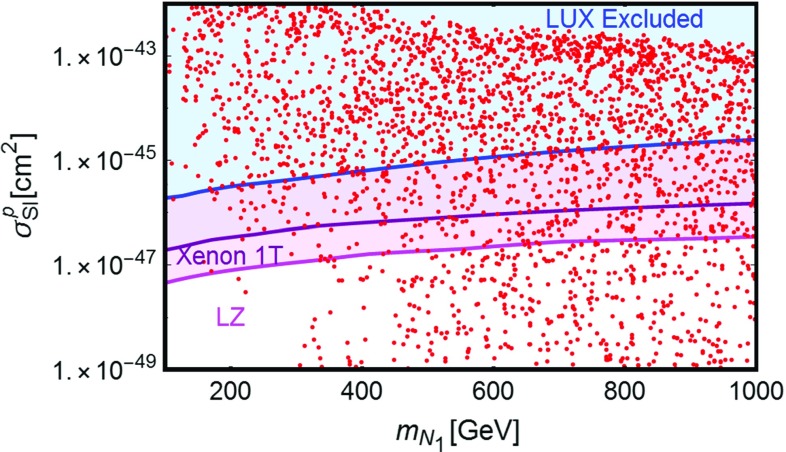
Model points satisfying the correct DM relic density and passing EWPT, perturbativity and unitarity constraints, in the bidimensional plane $(m_{N_1},\sigma _{SI})$. The *blue region* is excluded by current limits by LUX while the *purple and magenta* regions represent the reach of Xenon1T and LZ

The results of our analysis have been again reported, in Fig. 12, in the bidimensional plane $(m_{N_1},\sigma _\mathrm{SI})$. Similarly to the case of the single Higgs doublet scenario, many points, especially at lower values of the DM mass, are excluded by LUX. Viable model configurations nevertheless exist, already for DM masses of the order of 150 GeV. We notice in particular the presence of points lying beyond the reach of even next generation 1 Ton facilities like XENON1T and LZ. This is because, for these configurations, the relic density is achieved through the annihilations into $H^{\pm }H^{\mp }$ and $H^{\pm }W^{\mp }$ final states, relying on the couplings $y_{h,H}^{E_{L,R}}$, so that very small values of the neutral Yukawa couplings can be taken (as pointed above, in the presence of a single family of VLLs, large deviations from the custodial limit are allowed provided suitable assignments of the masses of the Higgs states.)

### Impact on LHC

In this section we will discuss the impact on LHC phenomenology of the scenario under investigation. In the subsections below we will provide an overview of the possible relevant processes, which currently are (and will be probed in the near future) by the LHC. These are distinguished in three categories: production of Higgs states and decay into SM fermions; production of the Higgs states and decay into gauge bosons, especially photons; direct production of VLLs. VLLs are directly involved only in the last two categories of collider signals; it is nevertheless important to consider as well limits/prospects from the first category of processes since they put constraints on the masses of Higgs states and on $\tan \beta$ which can, in turn, reduce the viable parameter space for DM.

Among this rather broad variety of signals we will pay particular attention to the diphoton production. It arises from the resonant production, and subsequent decay into photon pairs, of the neutral Higgs states. The VLL couplings entering in this process are the Yukawa couplings $y_{h,H}^{E_{L,R}}$. These couplings control the annihilation cross sections into $W^{\pm }H^{\mp }$ and $H^{\pm } H^{\mp }$ final states, which mostly account for the DM relic density in the high mass regime; furthermore, they are influenced, through the *S* / *T* parameters, by the values of the neutral couplings $y_{h,H}^{N_{L,R}}$, which are in turn strongly constrained by DM phenomenology.

As a further simplification we will consider the CP-even Higgs state *A* as the only candidate for a diphoton resonance. As will be explicitly shown in the following, this condition can be achieved by imposing a specific relation between the VLF Yukawa couplings, so as to minimize the impact of VLLs on the effective couplings between the CP-even state *H* and photons and, at the same time, maximize their impact on the effective $A\gamma \gamma$ coupling. This relation will allow one to reduce the number of free parameters. This choice is also motivated by the fact that the production cross section $pp \rightarrow A$ of the CP-odd state is, at parity of masses, bigger than the corresponding one of the CP-even state *H*. For the specific case of the diphoton production, as already pointed out, a further enhancement is achieved by a specific choice of the masses of the charged VLLs. As a consequence, focusing on the CP-odd Higgs *A* allows one to obtain conservative limits which can be straightforwardly extended to the CP-even *H*.

Despite these simplifications, there is still a broad variety of factors which influence the collider phenomenology of a diphoton resonance. We thus summarize below the most relevant cases, basically distinguished by the value of $\tan \beta$:

- *Low* $\tan \beta$, i.e. $\tan \beta =1-7$: The neutral Higgs states are mostly produced through gluon fusion. Irrespective of the type of couplings with the SM fermions (see Table 1), the top coupling to the heavy scalars is the dominant among the ones with SM fermions. This last coupling determines almost entirely the production cross sections of the processes $pp \rightarrow A/H$. The *H* / *A* resonances would then dominantly decay into $\bar{t} t$, or into a lighter neutral scalar (whether kinematically allowed) and a gauge boson,^14^ except for the case of sizable branching fractions of decay into charged and neutral VLLs (an important branching fraction into the DM would be nevertheless in strong tension with constraints from DM searches). In particular, for $\tan \beta =1$, one can have very large, $\Gamma /M \sim 5$–10%, decay width, given essentially by decays into $\bar{t} t$. The observation of $t \bar{t}$ resonances would be an interesting complementary signature of an eventual diphoton resonance. Searches of this kind of signals have been already performed at LHC Run I [85, 86]. The gluon–gluon fusion (ggF) mechanism can provide production cross sections close to the experimental sensitivity only for $\tan \beta \simeq 1$, while for increasing values of $\tan \beta$ it gets rapidly suppressed.
- *Moderate* $\tan \beta$, i.e. $\tan \beta =10-20$: While gluon fusion is still the most relevant production process, in a 2HDM with enhanced $\xi ^d_{H,A}$ (type-II and lepton-specific), a sizable contribution arises also from $b \bar{b}$ fusion. Regardless of the type of the 2HDM, the couplings between neutral resonances and SM fermions are suppressed, with respect to the previous scenario, so that they feature rather narrow width, unless sizable contributions arise from decays into VLLs (for $\tan \beta \gtrsim 5$ unitarity and perturbativity constraints favor a degenerate Higgs spectrum.). Large cross sections for the process $pp \rightarrow A/H \rightarrow \tau \tau$ are expected in a 2HDM with enhanced $\xi ^l_{H,A}$, i.e. type-II and flipped. Corresponding LHC searches [87, 88] give already strong limits, such that values of $\tan \beta$ above 10 are already excluded for $m_{A,H} < 500\,\text{ GeV }$.
- *High* $\tan \beta$, i.e. $\tan \beta \simeq 50$: This regime occurs only for the type-I and flipped 2HDM since the other cases are essentially ruled out, for masses of the neutral Higgses below approximately 1 TeV, by the limits from $pp \rightarrow A/H \rightarrow \tau \bar{\tau }$. Two rather different scenarios correspond to these two types of 2HDM. In the flipped model the *A* / *H* Higgs have enhanced couplings with *b*-quarks, implying $b \bar{b}$-fusion as dominant production process and, possibly, a large decay width dominated by the $b \bar{b}$ final state. In the case of the type-I 2HDM the neutral Higgses are “fermiophobic”, since all their couplings to the SM fermions are suppressed by a factor $1/\tan \beta$. Unless the decays into VLLs are relevant, we have very narrow widths, even $\Gamma _{H,A}/m_{H,A} \sim 10^{-2}$, and a strong enhancement of the decay branching fraction into photons.

In the following subsections we will provide an overview, for the scenarios depicted above, of the possible relevant LHC signals and the corresponding constraints/prospects of detection. We have indeed identified some relevant subsets among the parameter points providing the correct DM relic density and in agreement with theoretical constraints. We have first of all considered a set of points in the low, namely 1–5, $\tan \beta$ regime (although we will mostly refer to type-I 2HDM, the various 2HDM realizations do not substantially differ in this regime, as already pointed out). To these we have added three subsets, characterized by $10 \le \tan \beta \le 40$, for, respectively, type-I, type-II and lepton-specific couplings of the 2 Higgs doublets with the SM fermions. Two subsets at $\tan \beta =50$, corresponding to type-I and flipped realizations, have been finally included.

For our study we have adopted the cross sections provided by the LHC Higgs Cross Section Working Group [89], which have been produced with SusHi 1.4.1 [90]. More specifically, for the 2HDM types with enhanced bottom quark couplings to heavy scalars (type-II and flipped), we have taken the $gg/\bar{b}b$ fusion cross sections calculated for the *h*MSSM [91, 92]. For the remaining two realizations, namely type-I and lepton-specific 2HDMs, regardless of the value of $\tan \beta$, the only important production mechanism is *gg* fusion, since $\bar{b}b$ fusion is suppressed not only by the lower bottom quark luminosity, but also by the $\bar{b}bA/H$ couplings, which scale as $1/\tan \beta$. Therefore, as both top and bottom quark couplings to the heavy scalars are proportional to $1/\tan \beta$ for type-I and lepton-specific 2HDMs, it follows that the effective *ggA* / *H* couplings have a similar behavior. Consequently, for these two realizations, we evaluated the *gg* fusion cross sections by simply taking the *h*MSSM *gg*F cross section for $\tan \beta = 1$ and rescaling it by $1/\tan ^2\beta$.

#### $A/H \rightarrow \bar{f} f$

We will start our analysis by considering the production processes $pp \rightarrow \bar{f} f$.

**Fig. 13:**
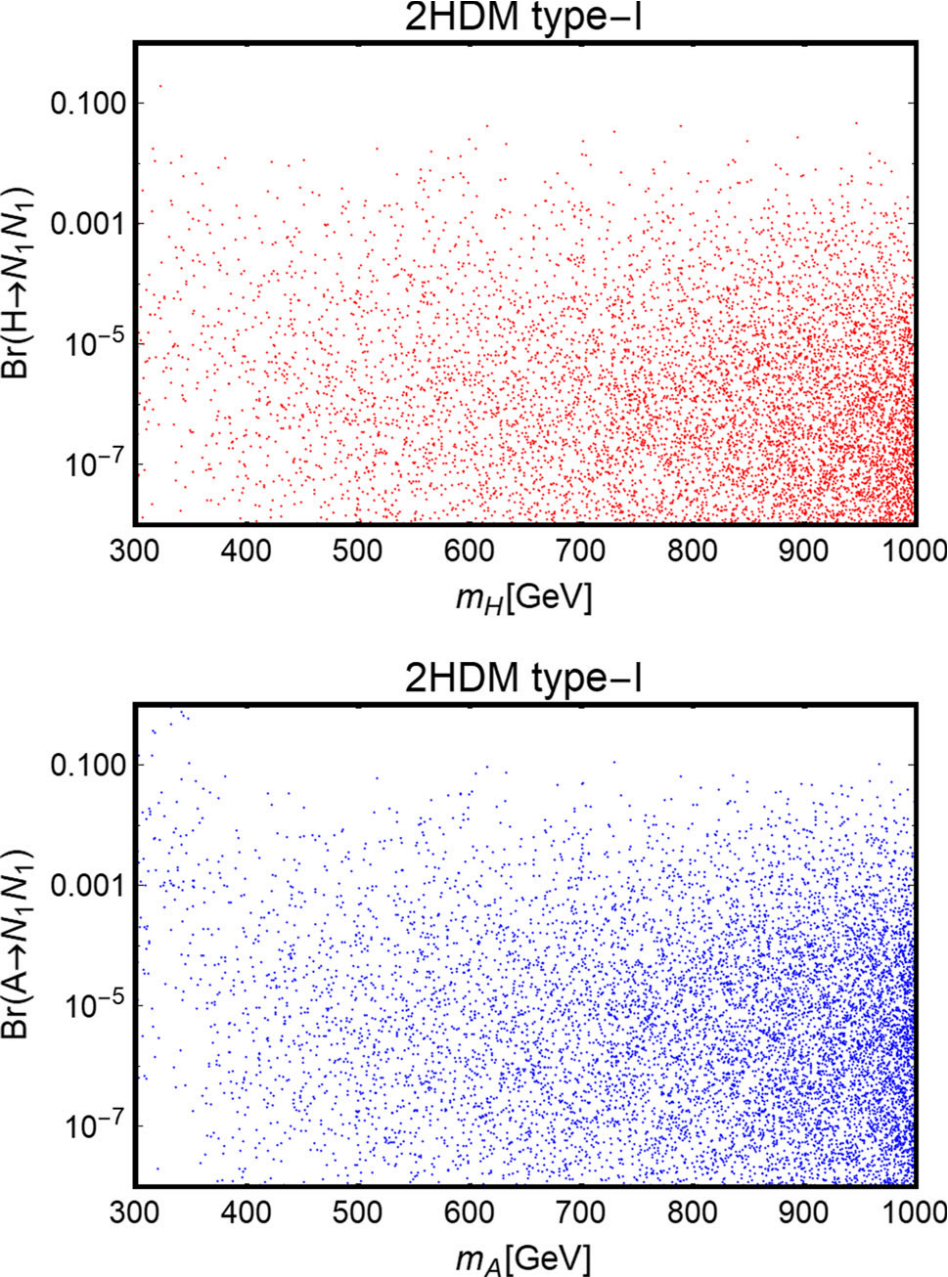
Decay branching ratios of the heavy CP-even (*top panel*) and CP-odd (*bottom panel*) scalar into $N_1 N_1$, as function of their masses

Their phenomenology is virtually identical to the pure 2HDM case. Indeed, being singlets under *SU*(3), the VLF do not modify the gluon fusion production vertex; furthermore, limits from DM phenomenology disfavor a sizable branching fraction of decay of the Higgs states into VLLs. For the case of DM this is easily understood by considering the strong limits from DM direct detection which require very suppressed couplings. A numerical check is provided on Fig. 13 for the case of type-I 2DHM (the outcome would be analogous also for the other types of 2HDM).

Here we have reported the branching ratios of decay into DM pairs of the *H* and *A* bosons for model points, generated through a parameter scan over the ranges illustrated in the previous section, featuring a DM scattering cross section below the current limit by the LUX experiment. The figure clearly evidences typically suppressed or even negligible values for these branching fractions. As shown in the bottom panel of the figure, a very small fraction of points for which $Br(A \rightarrow N_1 N_1)>10$ $\%$ is nevertheless present. These points correspond to $m_A \le 2 m_t$; as a consequence even for the low couplings imposed by DD constraints, the “invisible” branching fraction of the CP-odd Higgs can be comparable with the ones into SM fermions since, in absence of the $\bar{t} t$ channel, the latter are similarly suppressed by the Yukawa couplings (a further suppression is expected due to the fact that the couplings with the SM fermions are all proportional to $1/\tan \beta$. This result is specific of the type-I configuration. In other scenarios $\tan \beta$ enhancement of the couplings of the *A* with bottom and $\tau$ fermions instead occurs). On the contrary we see no points with $Br(A \rightarrow N_1 N_1)>10$ $\%$ when the decay into top pairs is kinematically accessible.

The couplings of the *H* / *A* bosons with the heavier VL neutrino and with the two VL electrons are, on the contrary, not directly constrained by direct detection and in principle could allow for sizable decay branching fractions. However, in two of the pinpointed scenarios for the correct DM relic density, i.e. s-channel resonances and annihilations into heavy Higgses, these decay processes are kinematically forbidden. Furthermore the coannihilation scenario is as well contrived for what regards collider prospects. We will then leave it aside for the moment and postpone a dedicated discussion to Sect. 3.5.4.

Since the branching fractions of the Higgses decaying into fermions depend on the masses of the final state fermions themselves, sizable signals can be achieved only for $\bar{t} t$, $\tau \tau$ and $\bar{b} b$ final states. The observation of the latter is substantially precluded by huge SM backgrounds so only $\bar{t} t$ and $\tau \tau$ feature observational prospects. Tau pair searches can probe type-II 2HDMs at moderate-to-high $t_{\beta }\gtrsim 5$, depending on the value of $m_A$, since in this case we have an enhancement of the $\tau$ Yukwawa coupling to *A*, $\xi ^{\tau }_A = t_{\beta }$. In a complementary manner, $t\bar{t}$ searches provide a discovery avenue for small values of $t_{\beta }$, typically $\lesssim$3 [93–96], for any type of 2HDM. However, looking for heavy scalars decaying into top quark pairs is challenging from the experimental point of view, since the interference between the signal and the SM background can give rise to non-trivial dip-peak structures in the $\bar{t}t$ invariant mass spectrum, which get smeared after binning, thus reducing the visibility of a potential “bump” [96, 97]. We also mention that the search for scalar resonances lighter than 500 GeV decaying to $\bar{t}t$ pairs is not possible, as the *t* and $\bar{t}$ quark are not boosted enough, the selection cuts thus being inefficient.

**Fig. 14:**
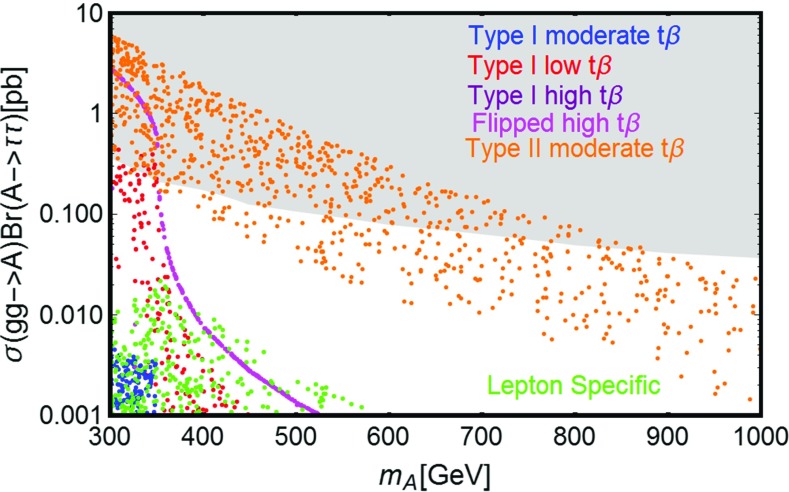
Production cross section for the process $pp \rightarrow \bar{\tau }\tau$ for the set of models with viable relic density. The *colors* distinguish the type of 2HDM realizations. The *gray region* is excluded by current limits [87, 88]

We have reported in Fig. 14 the $\tau \tau$ production cross section for the model points passing theoretical and DM constraints, distinguishing, with different colors, the various 2HDM scenarios depicted above. As already stated, current LHC constraints are mostly efficient in the 2HDM-II. They can nevertheless also exclude low values of $m_A$ for other 2HDM realizations.

**Fig. 15:**
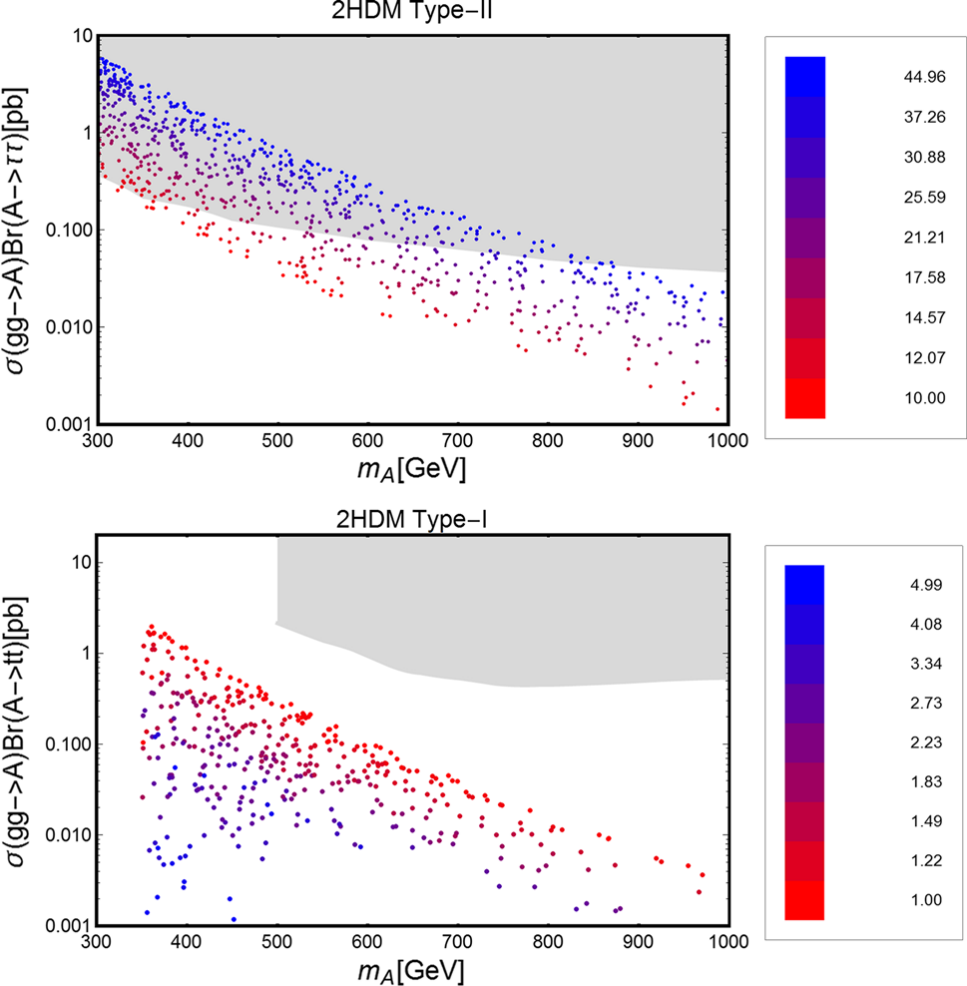
*Upper panel* $pp \rightarrow \bar{\tau }\tau$ cross section for type-II 2HDM. *Lower panel* $pp \rightarrow \bar{t} t$ for 2HDM type-I realizations in the low $\tan \beta$ regime. In both plots the points follow a color code according to the value of $\tan \beta$. The *gray regions* are already experimentally excluded

We have then focused, on the upper panel of Fig. 15, on the 2HDM-II case, highlighting the dependence of the collider limits on the value of $\tan \beta$. As evident, values above 20 are excluded for $m_A$ up to 1 TeV. A similar exercise has been performed on the lower panel of Fig. 15 for the case of the $pp \rightarrow A \rightarrow \bar{t} t$ process, in the scenario of very low $\tan \beta$. As evident, all the points lie below current experimental sensitivity. Only the points with $\tan \beta \sim 1$ lie close enough to the experimental sensitivity in order to be probed in the near future.

#### Diphoton signal

In this subsection we will investigate in more detail the prospects for observing a diphoton signal. The corresponding cross section can be schematically written as

50 $$\begin{aligned}&\sigma (pp \rightarrow \Phi \rightarrow \gamma \gamma )=\sigma (pp \rightarrow \Phi ) Br(\Phi \rightarrow \gamma \gamma ),\nonumber \\&\Phi =H,A, \end{aligned}$$

with

51 $$\begin{aligned} Br(\Phi \rightarrow \gamma \gamma ) \propto |\mathcal {A}_\mathrm{SM}^\Phi +\mathcal {A}_{H^{\pm }}^\Phi +\mathcal {A}_\mathrm{VLL}^\Phi |^2, \end{aligned}$$

where $\mathcal {A}_\mathrm{SM}^\Phi$, $\mathcal {A}_{H^{\pm }}^\Phi$ and $\mathcal {A}_\mathrm{VLL}^\Phi$ represent, respectively, the loop induced amplitudes by SM fermions, charged Higgs (only present for the CP-even state *H*) and VLLs.

The contribution associated to the VLLs can be written as

52 $$\begin{aligned} \mathcal {A}_\mathrm{VLL}^{\Phi } = \sum _{i=1}^2 \frac{v \left( \mathcal {C}_E^{\Phi }\right) _{ii}}{m_{E_i}} A_{1/2}^{\Phi } (\tau _{E_i}), \end{aligned}$$

where we have used the definition

53 $$\begin{aligned} \mathcal {C}_E^{\Phi } = U_L^E \cdot \mathcal {Y}_E^{\Phi } \cdot ( U_R^E )^{\dagger }. \end{aligned}$$

The Yukawa couplings between the VLLs and the heavy Higgs states are given by

54 $$\begin{aligned} \mathcal {Y}^H_N = \frac{1}{\sqrt{2}} \left( \begin{array}{cc} 0 &{} y_H^{N_L} \\ y_H^{N_R} &{} 0 \end{array}\right) , \quad \mathcal {Y}^H_E = \frac{1}{\sqrt{2}} \left( \begin{array}{cc} 0 &{} y_H^{E_L} \\ y_H^{E_R} &{} 0 \end{array}\right) , \end{aligned}$$

for the heavy CP-even scalar *H* and

55 $$\begin{aligned} \mathcal {Y}^A_N = \frac{1}{\sqrt{2}} \left( \begin{array}{cc} 0 &{} -y_H^{N_L} \\ y_H^{N_R} &{} 0 \end{array}\right) , \quad \mathcal {Y}^A_E = \frac{1}{\sqrt{2}} \left( \begin{array}{cc} 0 &{} y_H^{E_L} \\ -y_H^{E_R} &{} 0 \end{array}\right) , \end{aligned}$$

for the CP-odd scalar *A*.

A general analytical expression for Eq. (52) would be rather involved. We will, however, consider two simplifying assumptions. First of all, in order to avoid dangerous contributions to the decay branching fraction into photons of the SM-like Higgs we will set, as done before, $y_h^{E_R}=0$. Note that, especially in the case of heavier VLLs, one can relax this assumption, since the $h\rightarrow \gamma \gamma$ signal strength is currently measured with only $\sim 10-20$ $\%$ accuracy; nevertheless, for simplicity, we will take $y_h^{E_R}=0$. Furthermore, we will assume $M_E=M_L$, such that the mass matrix for the charged VLLs simplifies to^15^

56 $$\begin{aligned} \mathcal {M}_E = \left( \begin{array}{cc} M_E &{} v' y_h^{E} \\ 0 &{} M_E \end{array}\right) . \end{aligned}$$

Knowing that neither the sign of $M_E$ nor the one of $y_h^{E}$ are physical (both signs can be absorbed via a field redefinition), we will consider only positive values for these parameters. Thus, the eigenmass splitting reads

57 $$\begin{aligned} m_{E_2} - m_{E_1} = v' y_h^E, \end{aligned}$$

with $M_E = \sqrt{m_{E_1} (m_{E_1} + v' y_h^E)}$ fixed in order to give $m_{E_1}$ as the lowest eigenvalue. Under these assumptions the heavy scalar loop amplitudes can be written as

58 $$\begin{aligned}&\mathcal {A}_\mathrm{VLL}^H = \frac{-v'}{2 m_{E_1} + v' y_h^E}\nonumber \\&\quad \times \biggl \lbrace y_H^{E_L} \left[ A_{1/2}^H (\tau _{E_1}) - A_{1/2}^H (\tau _{E_2}) \right] \nonumber \\&\quad + y_H^{E_R} \left[ \frac{ m_{E_1} + v' y_h^E}{m_{E_1}} A_{1/2}^H (\tau _{E_1})- \frac{ m_{E_1} }{m_{E_1} + v' y_h^E } A_{1/2}^H (\tau _{E_2}) \right] \biggr \rbrace , \end{aligned}$$

59 $$\begin{aligned}&\mathcal {A}_\mathrm{VLL}^A = \frac{-v'}{2 m_{E_1} + v' y_h^E}\nonumber \\&\quad \times \biggl \lbrace y_H^{E_L} \left[ A_{1/2}^A (\tau _{E_1}) - A_{1/2}^A (\tau _{E_2}) \right] \nonumber \\&\quad - y_H^{E_R} \left[ \frac{ m_{E_1} + v' y_h^E}{m_{E_1}} A_{1/2}^A (\tau _{E_1}) - \frac{ m_{E_1} }{m_{E_1} + v' y_h^E } A_{1/2}^A (\tau _{E_2}) \right] \biggr \rbrace . \end{aligned}$$

To improve the detection potential of the heavy scalars decaying into diphotons, one should maximize the value of $$\mathcal {A}^A_\mathrm{VLL}$$. This task is completed by taking opposite signs for the $$ y_H^{E_R}, y_H^{E_L}$$ couplings. We can thus reduce the number of free couplings by setting $$ y_H^{E_R} = - y_H^{E_L} \equiv y_H^E$$. In this setup the *H* and *A* loop amplitudes become

60 $$\begin{aligned} \mathcal {A}^H_\mathrm{VLL} =&\frac{-v'^2 y_h^E y_H^E}{m_{E_1} (2 m_{E_1} + v' y_h^E)} \biggl [ A_{1/2}^H (\tau _{E_1})\nonumber \\&\quad + \frac{m_{E_1}}{m_{E_1} + v' y_h^E} A_{1/2}^H (\tau _{E_2})\biggr ], \end{aligned}$$

61 $$\begin{aligned} \mathcal {A}^A_\mathrm{VLL}&= \frac{v' y_H^E}{m_{E_1}} \left[ A_{1/2}^A (\tau _{E_1})- \frac{m_{E_1}}{m_{E_1} + v' y_h^E} A_{1/2}^A (\tau _{E_2})\right] . \end{aligned}$$

Note that, in the case where both $E_{1,2}$ mass eigenstates are much heavier than the scalar masses, i.e. $\tau _{E_{1,2}} \rightarrow 0$, the CP-even and CP-odd amplitudes differ only through the loop form factor:

62 $$\begin{aligned} \mathcal {A}^{A/H}_\mathrm{VLL} \simeq \frac{\pm v'^2 y_h^E y_H^E}{m_{E_1} ( m_{E_1} + v' y_h^E)} A_{1/2}^{A/H} (0). \end{aligned}$$

However, in the case where $A_{1/2}^A (\tau _{E_1})$ dominates over the second term in the brackets from Eq. (61), which happens, for example, if $m_{E_1}\simeq m_A/2$ and $m_{E_2} \gg m_{E_1}$, the CP-odd amplitude is indeed maximized: $\mathcal {A}^A_\mathrm{VLL} \propto \frac{v'}{m_{E_1}}$, whereas $\mathcal {A}^H_\mathrm{VLL} \propto \frac{v'^2}{m_{E_1}m_{E_2}}$.

**Fig. 16:**
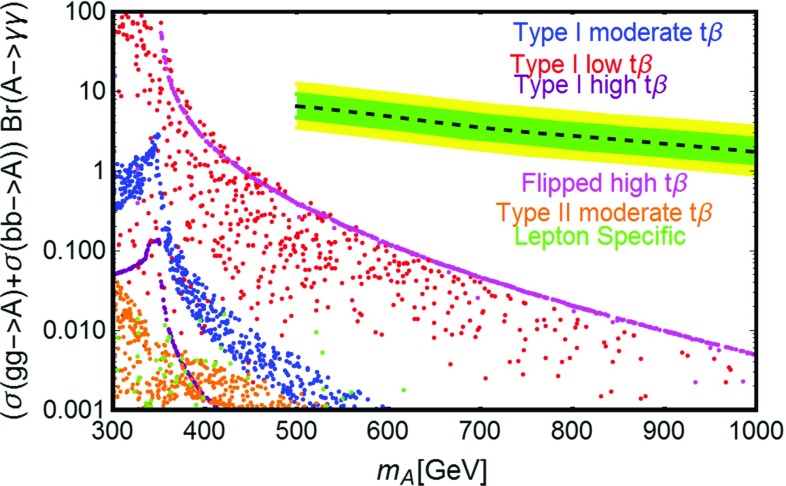
Expected diphoton cross section, as function of $m_A$ for the model points featuring the correct DM relic density and pass constraints from EWPT, perturbativity and unitarity. The *red points* refer to type-I couplings of the Higgs doublets while the *blue ones* to the other type of couplings considered in this work

We have reported, and confronted with the current experimental limits [98], in Fig. 16 the predicted cross section for $pp \rightarrow A \rightarrow \gamma \gamma$, for the model points providing viable DM candidates. We have distinguished between the different regimes described in the previous subsection, identified by the type of interactions with the fermions and by the value of $\tan \beta$. As evident, the most promising scenarios are the ones corresponding to low $\tan \beta$ and to $\tan \beta \sim 50$ for the flipped 2HDM. These scenarios correspond, indeed, to the configurations which maximize the production vertex of the resonance: as already emphasized, for $\tan \beta \sim 1$ the gluon fusion process is made efficient by the coupling with the top quark, while for $\tan \beta \sim 50$ the production cross section is enhanced by *b*-fusion. In the other type-I regimes, the cross section quickly drops with the value of $\tan \beta$.

In all the regimes considered the diphoton cross section lies below the current experimental sensitivity; the deviation from experimental sensitivity quickly reaches several orders of magnitude as the value of $m_A$ increases. A signal in diphoton events would be hardly observable, even in future luminosity upgrades, for $m_A \gtrsim 700\,\text{ GeV }$. The reason of this outcome mostly lies in the fact that the size of the Yukawa couplings of the charged VLLs are limited from above by the requirement of consistency under RG evolution and, only for $y_h^{E_L}$, by EWPT. As a consequence, no sensitive enhancement of the diphoton production cross section, with respect to the 2HDM without VLLs, is actually allowed. We notice, in addition, that in order to comply with limits from DM phenomenology, the VLLs should be typically heavier than the diphoton resonance. This translates in a further suppression of the VLL triangle loop contribution.

#### Other loop-induced processes

Given their quantum number assignments (and gauge invariance), VLLs also induce, at one loop, decays of *A* / *H* into $Z\gamma , ZZ, WW$, which can be probed at the LHC.

Among these processes, the cleanest signal is likely provided by the $Z\gamma$ channel. It is searched for in events with one photon and dijets or dileptons originating from the decay of the *Z*. Although the corresponding production rate is suppressed with respect to diphoton signals, the potential signal is particularly clean (i.e. low background), especially in the case of lepton final states. In the setup under investigation, the $A\rightarrow Z\gamma$ decay width, to a very good approximation, reads [45, 49]

63 $$\begin{aligned} \Gamma (A\rightarrow Z\gamma )&= \frac{\alpha g^2 m_A^3}{512 \pi ^4 v^2 c_W^2}\left( 1-\frac{m_Z}{m_A} \right) ^3\nonumber \\&\quad \,\times \left| \mathcal {A}_t^{AZ\gamma } + \mathcal {A}_b^{AZ\gamma } + \mathcal {A}_\mathrm{VLL}^{AZ\gamma } \right| ^2. \end{aligned}$$

The top-loop and bottom-loop amplitudes have simple expressions,

64 $$\begin{aligned} \mathcal {A}_{t,b}^{AZ\gamma } = N_c Q_{t,b} \, V_{t,b} \, \xi ^{t,b}_A A_{1/2}^A(\tau _{t,b}, \lambda _{t,b}), \end{aligned}$$

with $Q_f$ the electric charge of the SM fermion *f*, $V_f$ its vectorial coupling to the *Z* boson, and $\xi _A^{t,b}$ defined in Table 1. For the $A_{1/2}^A(\tau _i, \lambda _i)$ loop form factors, we use the same expressions as in Ref. [45], with $\tau _i \equiv \frac{m_A^2}{m_i^2}$ and $\lambda _i \equiv \frac{m_Z^2}{m_i^2}$.

Concerning the VLL $A\rightarrow Z\gamma$ loop amplitude, its general expression, which is again given in the appendix of Ref. [45] (denoted as $\tilde{\mathcal {A}}_f^{Z\gamma }$ there), is rather contrived, and will not be displayed here. However, for our particular choice of the charged VLL mass and pseudoscalar Yukawa matrices, it takes the simple form

65 $$\begin{aligned} \mathcal {A}_\mathrm{VLL}^{AZ\gamma }&= Q_e \, V_e \frac{v' y_H^E}{m_{E_1}} \nonumber \\&\quad \times \,\left[ A_{1/2}^A(\tau _{E_1}, \lambda _{E_1}) - \frac{m_{E_1}}{m_{E_1} + v' y_h^E} A_{1/2}^A(\tau _{E_2}, \lambda _{E_2}) \right] , \end{aligned}$$

with $Q_e = -1$ the electric charge of the VL electron and $V_e = -0.25 + s_W^2$ the vectorial coupling to the Z of the SM electron. One can see that, contrary to the general case, the diagrams with off-diagonal *A* and *Z* couplings to the VLFs vanish for our choice of parameters. Unfortunately, due to the smallness of $V_e \simeq 0.02$, our scenario does not produce a sizable modification to the $A\rightarrow Z\gamma$ decay channel with respect to the case of an ordinary 2HDM.

We also briefly comment on the case of the *WW* and *ZZ* decay channels. As the $A\rightarrow \gamma \gamma /Z\gamma$ processes, both $A\rightarrow ZZ$ and $A\rightarrow WW$ are loop-suppressed (*AWW* / *AZZ* vertices are forbidden at tree level by CP-invariance). Moreover, detection of such decays is challenging due to either (i) suppression by reduced branching ratios ($\mathrm{Br}(Z\rightarrow \ell ^+\ell ^-) \simeq 7$ $\%$, $\ell =e,\mu$) or (ii) final states that are difficult to reconstruct/disentangle from the background (hadronic decays of *W*, *Z* and leptonic decays of the *W*, $W\rightarrow \nu \ell$, which involve missing transverse energy). Therefore, we will not consider these channels as they are not as clean and/or competitive as the ones already discussed.

#### Direct production of VLLs

We conclude our overview of the collider phenomenology of the scenario under investigation by briefly commenting on possible direct searches of the VLLs. VLLs can be produced at LHC through the Drell–Yann processes [27, 99–101] $pp \rightarrow Z^{*}/\gamma ^{*} \rightarrow EE$, $pp \rightarrow Z^{*} \rightarrow NN$, and $pp \rightarrow W^{*} \rightarrow NE$.^16^ The results of corresponding LHC searches [105, 106] cannot be, nevertheless, applied to our case since they rely on the presence of a mixing with SM leptons. In our scenario, in order to guarantee the stability of the DM candidate, we have forbidden such a mixing by imposing a $\mathbb {Z}_2$ symmetry under which the VLL sector is odd and the SM is even (see next section). On the contrary, a possible collider signal would be represented by the production of $E_1 E_1$ or $N_2 E_1$ and their subsequent decay into DM, which can be tested in 2–3 charged leptons plus missing energy final state events. Searches of this kind have been performed in the context of supersymmetric scenarios [107–109]. In order to take into account possible constraints, we have imposed (ad exception of the coannihilation regime), in our scans, a lower limit on the mass of the lightest charged VLL of 300 GeV. Direct production of DM, through off-shell Z/h boson or on-shell heavy Higgses, can be instead hardly tested, through monojet searches, since constraints from DM direct detection imposes, in most of the phenomenologically viable parameter space (see discussion in the previous section) a negligible branching fraction of decay into DM pairs (see Fig. 13).

Another potentially interesting channel would be the production of a charged Higgs and its subsequent decay into $N_1 E_1$, followed by $E_1 \rightarrow N_1 W$. However, for most of the points providing the correct DM relic density and, at the same time, passing the DD constraints, we have $m_{H^{\pm }} < m_{N_1}+m_{E_1}$, so that production can occur only through off-shell charged Higgs. Furthermore, the dominant production modes of $H^{\pm }$ at the LHC, $gg \rightarrow t b H^{\pm }$ and $gb \rightarrow t H^{\pm }$, are phase-space suppressed by the top quark produced in association and typically have a low cross section. The *s*-channel production of a charged Higgs, $qq' \rightarrow H^{\pm }$ is not a valid option neither: even if the charged Higgs would be on-shell, the low Yukawa couplings of the initial state quarks renders such a process unobservable. For a more detailed discussion, we refer the reader to Ref. [27].

We close the section by commenting again on possible production of VLLs from decays of the neutral Higgses. As pointed out in Sect. 3.5.1, in the coannihilation scenario sizable branching fractions for the decays $H/A \rightarrow E_i E_i,\,i=1,2$ are not forbidden by limits from DM phenomenology. However, while $E_2$, having a sizable admixture of a $SU(2)_L$ doublet, almost always decays promptly into $E_1$ plus a *W* / *Z* / *h* boson (on or off-shell), the $E_1$ can decay only into a $N_1$ and two fermions through an off-shell *W*. This decay rate would be doubly suppressed by the very small coupling to the *W* of the mostly SU(2) singlet DM and by the phase space. Consequently, the $E_1$ state would be long-lived or even stable on collider scales.

### Constraints on the charged Higgs

Collider limits on the charged Higgs are mostly relevant for very light masses, namely $m_{H^{\pm }} < m_t$. In this case, light charged Higgses can be searched for in the $t \rightarrow H^{\pm } b$ decays, followed by $H^{\pm } \rightarrow c s$ or $H^{\pm } \rightarrow \tau \nu _\tau$. Searches for this processes have been performed both by ATLAS [110] and CMS [111, 112]. No sensitive variations in the top branching fractions with respect to the SM have been detected, disfavoring masses of the charged Higgs below 160 GeV. The ATLAS collaboration has performed searches for $H^{\pm }\rightarrow \tau \nu _\tau$ [113] also in the high mass regime, i.e. $m_{H^{\pm }} > m_t$, with the charged Higgs being produced in association with a top quark, i.e. through the process $g b \rightarrow t H^{\pm }$. The limits obtained, however, cannot yet constrain efficiently most of the 2HDM setups considered in this work (with the possible exception of the lepton specific 2HDM), since the $\tau \nu _\tau$ final state has a low branching fraction at high masses [114].

The mass of the charged Higgs can also be strongly constrained by low energy observables. As these bounds are determined by the value of $\tan \beta$, they are actually dependent on the type of 2HDM realization. For an extensive review we refer, for example, to Ref. [114]. We will instead summarize, in the following, the constraints relevant to our analysis.

We have first of all to consider loop-induced contributions to the $B\rightarrow X_s \gamma$ process. These depend on the coupling of the charged Higgs to *t*,*b* and *s* quarks. In the type-I and lepton-specific models, all the relevant couplings are suppressed by $1/\tan \beta$ and, hence, sizable constraints are obtained only for very low $\tan \beta$ [115]. Much stronger bounds are instead obtained in 2HDM-II, excluding masses of the charged Higgs up to order of 400 GeV [116–119], practically independent from the value of $\tan \beta$. A second relevant bound comes from the semileptonic decays of the pseudoscalar mesons, in particular $B(B \rightarrow \tau \nu )$. By requiring the ratio $r=B(B \rightarrow \tau \nu _\tau )/B(B \rightarrow \tau \nu _\tau )_\mathrm{SM}$ to be consistent with the experimental determination $r=1.56 \pm 0.47$ [120, 121], one obtains, only for the type-II 2HDM, a limit on the bidimensional $(m_{H^{\pm }},\tan \beta )$ plane which is relevant for $\tan \beta \gtrsim 20$.

### Stability of DM and flavor changing neutral currents

As already stated, our analysis has been mostly carried on a purely phenomenological basis. In this subsection we will nevertheless take some steps towards a more complete construction discussing some potential challenges in the model building, the stability of the DM and the suppression of FCNCs.

VLFs with the same quantum numbers of the SM fermions allow for yukawa coupling of one VLF, one SM fermion and one Higgs boson, responsible for a mass mixing which makes all the VLFs to decay into a SM fermion and a gauge or Higgs boson. In order that the lightest neutral VLF is a viable DM candidate this kind of mixing should be strongly suppressed or possibly forbidden. The simplest option to achieve this goal would be represented by the introduction of a $\mathbb {Z}_2$ symmetry, which we label $\mathbb {Z}_2^\mathrm{VLL}$, under which the VLLs are odd (with the SM states being instead even), so that the coupling originating with the mixing between the VLLs and the SM fermions would be actually forbidden.

Another potential challenge is represented by the presence of FCNCs. FCNCs induced by the coupling of the SM fermions with the Higgs doublets have been forbidden by assuming four specific configurations for these couplings. These can be realized by assuming suitable discrete symmetries. The type-I configuration realized by assuming a discrete symmetry $\mathbb {Z}_2^\mathrm{2HDM}$ such that $H_1 \rightarrow -H_1$ so that all the SM fermions are coupled to the $H_2$ doublet, while in the type-II configuration, also the right-handed d-quarks and right-handed leptons are odd under this $\mathbb {Z}_2$ symmetry so that they are coupled with the $H_1$ doublet. The lepton-specific and flipped configurations are similarly realized through suitable assignation of the $\mathbb {Z}_2$ charges for up-quarks, down-quarks and leptons. Possible UV completions for these 2HDM realizations have been studied e.g. [122–124].

The addition, to the mass spectrum, of VLFs, freely coupled to both Higgs doublets, provides a further potential source of FCNCs, induced at one-loop in this case. The determination of possible bounds for generic couplings of the VLFs, as considered here, is not in the purpose of this work. A possible simple solution would be represented by making also some of the VLFs odd under the $\mathbb {Z}_2^\mathrm{2HDM}$ symmetry. Drawing inspiration from the simple flavor conserving 2HDMs, where the SM quark doublet and the up-type right-handed quarks are, by convention, even under $\mathbb {Z}_2^\mathrm{2HDM}$, we choose the VLL doublet and the $N^{\prime }_{L,R}$ singlet VL neutrino (“up-type” VLL) to be, similarly, even under $\mathbb {Z}_2^\mathrm{2HDM}$. This leaves us with two possibilities for $E^{\prime }_{L,R}$:

1. $E^{\prime }_{L,R}$ is also even under $\mathbb {Z}_2^\mathrm{2HDM}$, meaning that all VLLs couple to $H_2$;
2. $E^{\prime }_{L,R}$ is odd under $\mathbb {Z}_2^\mathrm{2HDM}$, which implies that VL electrons couple to $H_1$, while VL neutrinos couple to $H_2$.

As evident from the discussion above, $\mathbb {Z}_2^\mathrm{2HDM}$ and $\mathbb {Z}_2^\mathrm{VLL}$ should be distinct symmetry groups since the VLLs have the same charge under $\mathbb {Z}_2^\mathrm{VLL}$ but different charges under $\mathbb {Z}_2^\mathrm{2HDM}$.

Once the two symmetries are imposed, the Higgs+VLL Lagrangian reads as follows (for simplicity, mass terms have been omitted since not relevant for the discussion):

66 $$\begin{aligned} -\mathcal{L}_{VLL}&= y_{N_R} \overline{L}_L \tilde{H_2} N_R^\prime + y_{N_L} \overline{N}^\prime _L \tilde{H_2}^\dagger L_R\nonumber \\&\quad + y_{E_R} \overline{L}_L H_i E^\prime _R + y_{E_L} \overline{E}^\prime _L H^\dagger _i L_R + \mathrm {h.c.}, \end{aligned}$$

where $i=1,2$ corresponds to the two cases mentioned above. After EWSB, the interactions lagrangian of the VL neutrinos with the neutral Higgs scalars is the same in the two cases and reads

67 $$\begin{aligned}&-\sqrt{2}\,\mathcal {L}_{\phi NN}= \begin{pmatrix} N_L^{\dagger }&N_L^{\prime \dagger } \end{pmatrix} \biggl [ h\begin{pmatrix} 0 &{} y_{N_R} s_{\beta } \\ y_{N_L} s_{\beta } &{} 0 \end{pmatrix}\nonumber \\&\quad + H \begin{pmatrix} 0 &{} -y_{N_R} c_{\beta } \\ -y_{N_L} c_{\beta } &{} 0 \end{pmatrix} + A \begin{pmatrix} 0 &{} -i \,y_{N_R} c_{\beta } \\ i \, y_{N_L} c_{\beta } &{} 0 \end{pmatrix} \biggr ] \begin{pmatrix} N_R \\ N_R^{\prime }; \end{pmatrix}\nonumber \\&\quad + \mathrm {h.c.} \end{aligned}$$

on the contrary, in the case of the VL electrons we distinguish the following two possibilities:

68 $$\begin{aligned}&-\sqrt{2}\,\mathcal {L}_{\phi EE}^{(1)}= \begin{pmatrix} E_L^{\dagger }&E_L^{\prime \dagger } \end{pmatrix} \biggl [ h\begin{pmatrix} 0 &{} y_{E_R} c_{\beta } \\ y_{E_L} c_{\beta } &{} 0 \end{pmatrix}\nonumber \\&\quad + H \begin{pmatrix} 0 &{} y_{E_R} s_{\beta } \\ y_{E_L} s_{\beta } &{} 0 \end{pmatrix} + A \begin{pmatrix} 0 &{} -i \,y_{E_R} s_{\beta } \\ i \, y_{E_L} s_{\beta } &{} 0 \end{pmatrix} \biggr ] \begin{pmatrix} E_R \\ E_R^{\prime } \end{pmatrix}\nonumber \\&\quad + \mathrm {h.c.}, \end{aligned}$$

69 $$\begin{aligned}&-\sqrt{2}\,\mathcal {L}_{\phi EE}^{(2)}= \begin{pmatrix} E_L^{\dagger }&E_L^{\prime \dagger } \end{pmatrix} \biggl [ h\begin{pmatrix} 0 &{} y_{E_R} s_{\beta } \\ y_{E_L} s_{\beta } &{} 0 \end{pmatrix}\nonumber \\&\quad + H \begin{pmatrix} 0 &{} -y_{E_R} c_{\beta } \\ -y_{E_L} c_{\beta } &{} 0 \end{pmatrix} + A \begin{pmatrix} 0 &{} i \,y_{E_R} c_{\beta } \\ -i \, y_{E_L} c_{\beta } &{} 0 \end{pmatrix} \biggr ] \begin{pmatrix} E_R \\ E_R^{\prime } \end{pmatrix}\nonumber \\&\quad + \mathrm {h.c.}. \end{aligned}$$

As regards the interactions of the VLLs with the charged Higgs we have

70 $$\begin{aligned}&-\mathcal {L}_{H^{\pm } NE}^{(1)} = H^+ \begin{pmatrix} N_L^{\dagger }&N_L^{\prime \dagger } \end{pmatrix} \begin{pmatrix} 0 &{} y_{E_R} s_{\beta } \\ y_{N_L} c_{\beta } &{} 0 \end{pmatrix} \begin{pmatrix} E_R \\ E_R^{\prime } \end{pmatrix}\nonumber \\&\quad + H^- \begin{pmatrix} E_L^{\dagger }&E_L^{\prime \dagger } \end{pmatrix} \begin{pmatrix} 0 &{} y_{N_R} c_{\beta } \\ y_{E_L} s_{\beta } &{} 0 \end{pmatrix} \begin{pmatrix} N_R \\ N_R^{\prime } \end{pmatrix}\nonumber \\&\quad + \mathrm {h.c.}, \\&-\mathcal {L}_{H^{\pm } NE}^{(2)} = H^+ \begin{pmatrix} N_L^{\dagger }&N_L^{\prime \dagger } \end{pmatrix} \begin{pmatrix} 0 &{} -y_{E_R} c_{\beta } \\ y_{N_L} c_{\beta } &{} 0 \end{pmatrix} \begin{pmatrix} E_R \\ E_R^{\prime } \end{pmatrix}\nonumber \\&\quad + H^- \begin{pmatrix} E_L^{\dagger }&E_L^{\prime \dagger } \end{pmatrix} \begin{pmatrix} 0 &{} y_{N_R} c_{\beta } \\ -y_{E_L} c_{\beta } &{} 0 \end{pmatrix} \begin{pmatrix} N_R \\ N_R^{\prime } \end{pmatrix}\nonumber \\&\quad + \mathrm {h.c.} \end{aligned}$$

The couplings introduced in this subsection can be related to the ones used in our analysis by reabsorbing a factor $s_{\beta }$ ($c_{\beta }$), in the case that $E_{L,R}$ ($E^{\prime }_{L,R}$) couples to $H_1$($H_2$), into the definitions of the VLL Yukawa couplings to the 125 GeV Higgs boson, *h*. For the VL neutrinos, the redefined couplings to the scalars would read

71 $$\begin{aligned} y_h^{N_{L,R}} \equiv y_{N_{L,R}} s_{\beta }, \quad y_H^{N_{L,R}} \equiv -y_{N_{L,R}} c_{\beta } = - y_h^{N_{L,R}} t_{\beta }^{-1}, \end{aligned}$$

whereas for the VL electrons we have

72 $$\begin{aligned} y_h^{E_{L,R}} \equiv y_{E_{L,R}} c_{\beta }, \quad y_H^{E_{L,R}} \equiv y_{E_{L,R}} s_{\beta } = y_h^{E_{L,R}} t_{\beta } \end{aligned}$$

73 $$\begin{aligned} y_h^{E_{L,R}} \equiv y_{E_{L,R}} s_{\beta }, \quad y_H^{E_{L,R}} \equiv -y_{E_{L,R}} c_{\beta } = -y_h^{E_{L,R}} t_{\beta }^{-1}. \end{aligned}$$

in the cases of, respectively, couplings with $H_1$ and $H_2$.

We then notice that, contrary to the case where the VLLs couple to both scalar doublets, $t_{\beta }$ plays now a role also in the VLL sector. More specifically, one finds, in the VLL couplings to *H*, *A* (relative to their couplings to *h*),the same type of $t_{\beta }^{-1}$ suppression or $t_{\beta }$ enhancement as for the SM fermions.

**Fig. 17:**
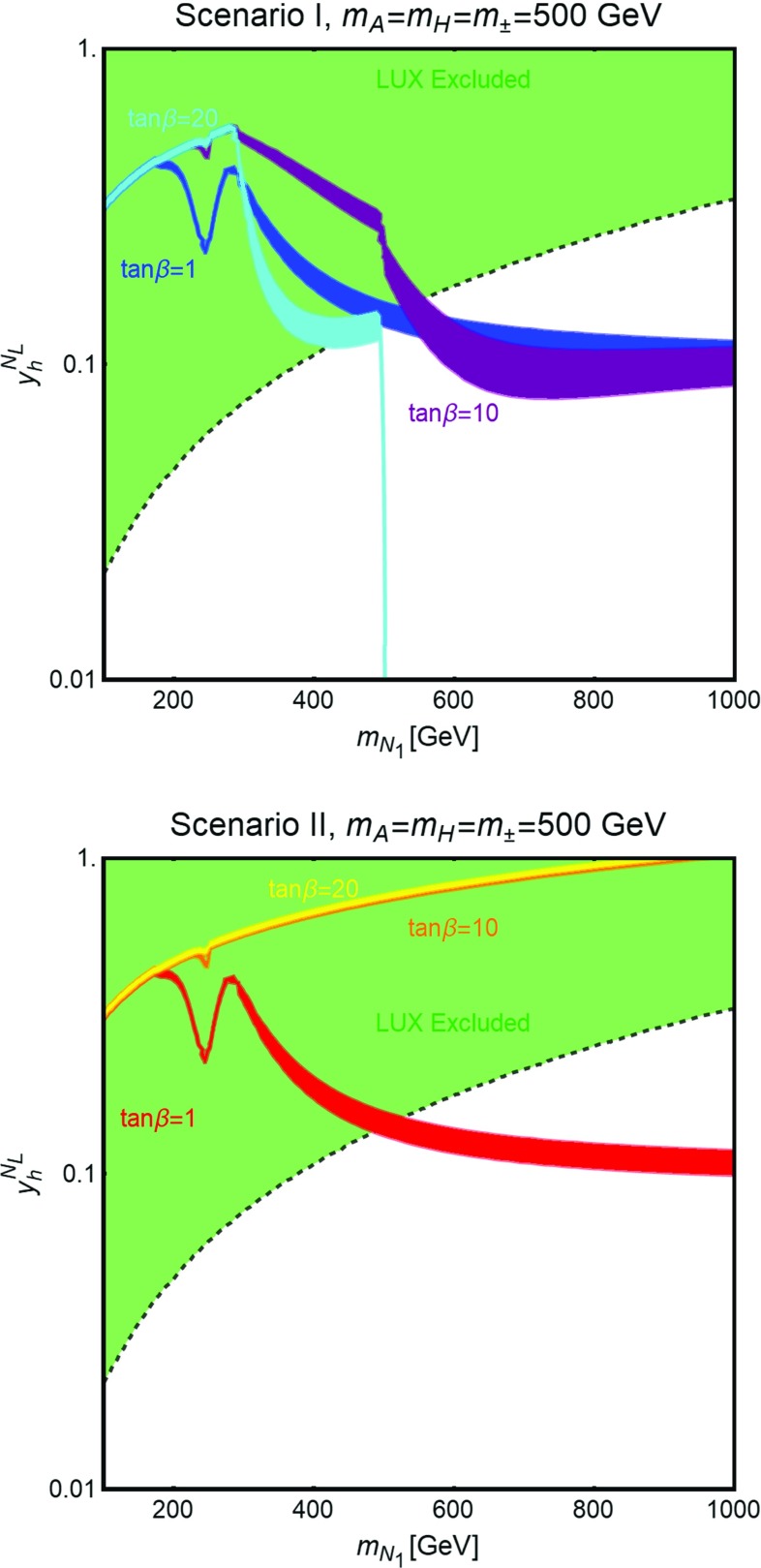
Main constraints from dark matter phenomenology, i.e. in the two proposed scenarios for flavor conserving VLF sector

The impact of this feature on DM phenomenology has been sketched in Fig. 17. Here we have reported in the two panels the isocontours of the correct DM relic density, as well as the excluded region by LUX, in the bidimensional plane $(m_{N_1},y_h^{N_L})$ (we have used the relations above to adopt the same variables as the rest of the text. We have also assumed $y_h^{N_R}=0$), and for three values of $\tan \beta$, namely 1,10 and 20, while keeping fixed $y_h^{E_L}=0.1$, $y_h^{E_R}=0$. For numerical convenience we have fixed $M_L=M_E=2 M_N$ rather than to a constant value, as the analogous plots in Sect. 2.4. This implies a different morphology for the LUX excluded region, with respect to the one shown in Fig. 8. This is because keeping $M_L$ and $M_E$ fixed to a constant value, rather than considering a constant ratio with $M_N$, changes the behavior of the angles $\theta _N^{L,R}$ with $y_h^{N_L}$ (see Eq. 14). The choice of the constant ratio implies, in particular, stronger bounds at low DM masses.

The more constrained, with respect to general case discussed in the rest of the text, structure of the couplings influences the scenarios for the correct relic density, i.e. s-channel resonances and annihilation into heavy Higgs bosons in the following way. The relation between the couplings $y_h^{N_L}$, $y_H^{N_L}$ tends to disfavor the case of resonant annihilations since they make the constraints from DD stronger with respect to the case in which these two couplings can be regarded as independent. The effectiveness of these constraints increases with $\tan \beta$ since the couplings of the DM with the *H* and *A* bosons are now suppressed as $1/\tan \beta$. The regime of annihilations into heavy Higgs bosons is perfectly viable in the case from Eq. (73) (“Scenario I”) where the coupling $y_H^{E_L}$ can be even enhanced at high $\tan \beta$ (in particular for $\tan \beta =20$ this annihilation is so strong that the DM results are underabundant in the range of the parameters reported in the plot). More contrived is instead the case from Eq. (74) (“Scenario II”) where DM annihilation into $H^+H^-$ is suppressed at high $\tan \beta$, thus increasing the tension with DD constraints. The light DM regime is, instead, negligibly affected since the relic density is mostly determined by the couplings of the DM with the *W* and *Z* boson which depend only on $y_h^{N_L}$ and $y_h^{E_L}$. For the same reason, the constraints from direct detection do not change by varying $\tan \beta$.

### Summary of results

**Fig. 18:**
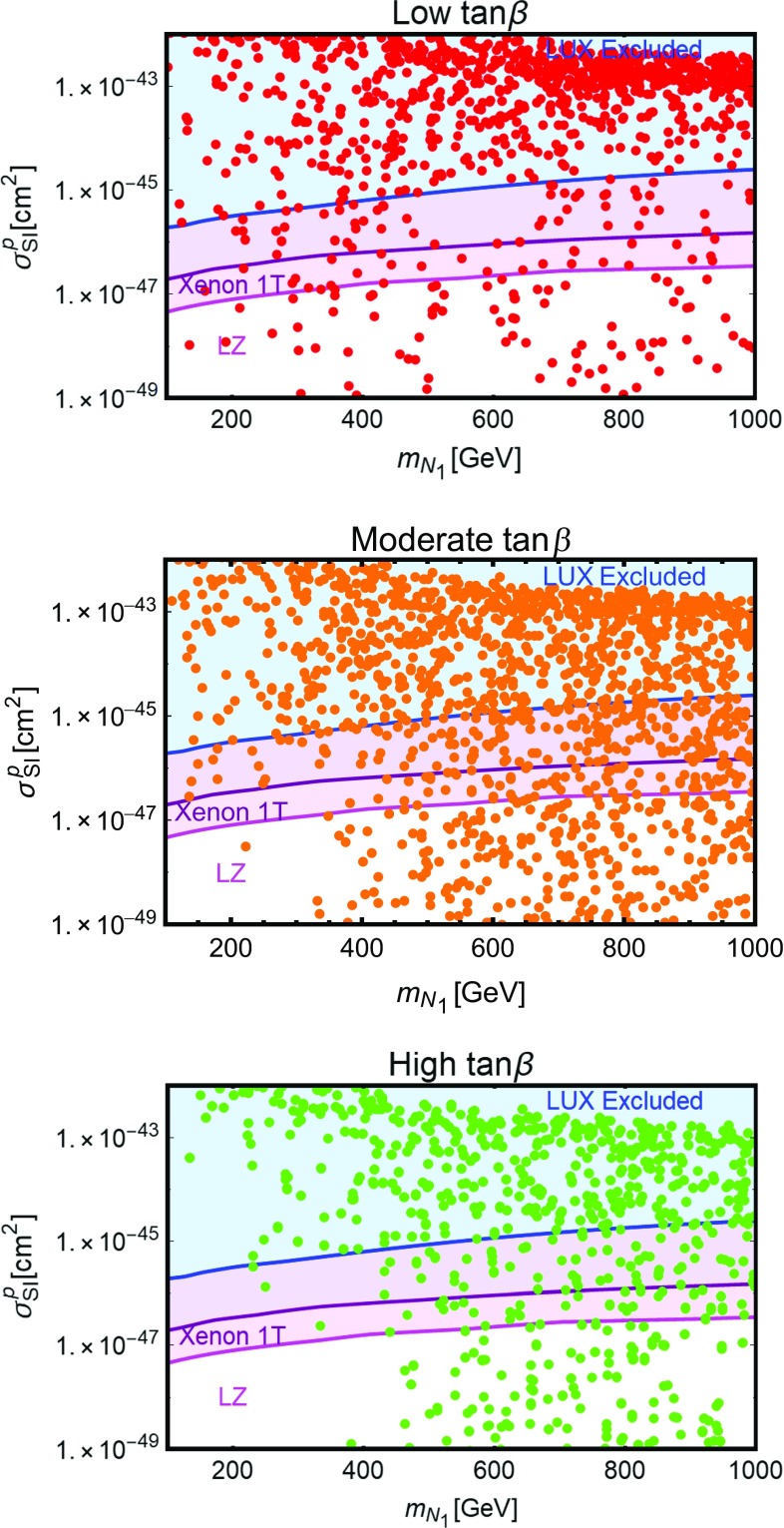
Summary plots including all the constraints discussed throughout this work. Each of the *three panels* of the figure refers to a different regime of values of $\tan \beta$ (see main text for details), indicated on the *top of each panel*

The results of our study are summarized in Fig. 18. Here we have put together all the results for DM phenomenology with theoretical constraints, i.e. scalar quartic couplings RGEs, EWPT constraints, limits from collider searches, mostly $H/A \rightarrow \tau \tau$, and constraints from low energy observables (for the latter we have adopted the limits on $(m_{H^{\pm }},\tan \beta )$ as reported in Refs. [115, 125]).

The three panels of Fig. 18 show, for three regimes of values of $\tan \beta$, i.e. low, moderate and heavy (see Sect. 2.5), in the $(m_{N_1},\sigma _{N_1 p}^\mathrm{SI})$ plane, the model points providing the correct DM relic density and satisfying the constraints listed above.

The results reported in Fig. 18 can be explained as follows. The distributions of the points in the three panels of the figure appear to be rather similar. As discussed in the text, under the assumption that the VLL can couple with both Higgs doublets, the dependence on $\tan \beta$ of the couplings of the DM is reabsorbed in the definition of the couplings themselves. We notice nevertheless that light DM masses, i.e. lighter than approximately 400 GeV become progressively disfavored as the value of $\tan \beta$ increases. DD limits are mostly evaded if the DM relic density is achieved either in correspondence of s-channel resonances or by annihilations involving heavy Higgs bosons, in particular the charged ones, as final states. The former possibility becomes increasingly contrived at higher values of $\tan \beta$ because the reduced decay width of the *H* / *A* states requires a stronger fine tuning in the $|m_{N_1}-m_{A,H}/2|$ ratio (a possible exception would be represented by the flipped 2HDM at very high, i.e. ${\gtrsim } 45$, $\tan \beta$). This problem is partially overcome by considering high enough values of the masses of *H* and *A*. The case of the annihilations into heavy Higgs bosons is influenced by several aspects, according the configuration, i.e. type-I, type-II, lepton-specific or flipped, chosen for the couplings with SM fermions. The type-II configuration is excluded for $m_A$ below 400 GeV in the moderate $\tan \beta$ regime, and for considerably higher masses in the high $\tan \beta$ regime, by LHC searches in the $\tau \tau$ channel (cf. Fig. 14). Values of $m_A$ below 400 GeV are also excluded in the flipped configuration for high $\tan \beta$. These constraints also partially influence the other Higgs masses since for moderate/high $\tan \beta$ the constraints 34 and 35 and EWPT tend to favor a mass degenerate heavy Higgs spectrum. In the type-II model the mass of the charged Higgs is, nevertheless, individually constrained to be above approximately 400 GeV by constraints from low energy processes.

## Conclusions

In this work, we have performed an extensive study of the impact of the addition of a family of vector-like fermions, with suitable quantum numbers such as to provide a DM candidate, to the SM and to various types of 2HDMs.

The SM$+$VLLs realization is strongly constrained. The correct relic density implies too strong interactions with the *Z*-boson, ruled out by DM direct detection unless the DM, and hence in turn the whole spectrum of the new fermions, lies above the TeV scale.

Lower DM masses can instead be achieved in 2HDM realizations. Indeed, s-channel enhancement, in correspondence with the *H* / *A* poles, can provide the correct relic density even for a small hypercharge/SU(2) component of the DM. In addition, efficient DM annihilations can also be achieved, in the $H^{\pm } H^{\mp }$ and $W^{\pm } H^{\mp }$ final states. The corresponding cross section is not directly correlated with the DM DD cross section, such that it would be possible to evade current and even next future bounds. On the other hand, the DM relic density depends on the masses of the new Higgs states. Complementary constraints thus come from their experimental searches. Given their dependence on $\tan \beta$ the allowed parameter space actually depends on the type of couplings of the Higgs doublets with the SM fermions.

Type-II, and to lesser extent, flipped 2HDMs, are the most constrained since low values of $m_{H^{\pm }}$ (and in turn DM masses) are excluded by low energy observables. Moreover a large part of the type-II parameter space is excluded by limits from searches of $A/H \rightarrow \tau \tau$. Combining these constraints, DM masses below 400 GeV are strongly disfavored. For the other two 2HDM realizations, constraints from searches of extra Higgses are not yet competitive with DM constraints and lower DM masses are accessible.

Although the size of the Yukawa couplings of the charged VLLs can account for the correct DM relic density, it does not account for a significant enhancement of the diphoton production rates observable at colliders. This happens because the limits from EWPT and RGE forbid values greater than $\sim 1$ for thes couplings.

Moreover, the possibility of a direct observation of the VLLs appears similarly contrived in particular for what regards the DM. It could be indeed produced, in a 2HDM setup, by the decays of the neutral H/A or of the charged $H^{\pm }$ (in this case rather than pair DM production one would have production of one DM state in association with the lightest VL lepton $E_1$) or, alternatively through the production of virtual *h* / *Z* bosons. Concerning the DM candidate $N_1$, sizable couplings with the neutral Higgs bosons (as well as with the *Z* boson) are disfavored especially by DM direct detection constraints so that the corresponding decay branching fractions are suppressed (an exception of a small region parameter space for type-I 2HDM). In addition, in correspondence with two of the principally considered scenarios for the correct DM relic density, decays of neutral bosons into DM pairs are phase-space suppressed (e.g. in the s-channel resonance scenario) or even forbidden. The coupling between the DM, the lightest electrically charged VLL, and the charged Higgs is not required to be suppressed; however, the production of $N_1 E_1$ from decays of the charged Higgs is similarly disfavored since in most of the viable parameter space this decay is kinematically forbidden.

## Footnotes

1 Note that $\mu _{hZ\gamma }$ is also affected by the VLFs, but the uncertainties on this signal strength are too large to constrain the extended fermionic sector [47, 48].

2 Alternatively one could think about a cancellation between the contributions of the “up” and “down” sectors. In order to have a DM candidate we will consider, however, in this work the case that the up sector is made by electrically neutral states, so that they do not actually contribute to $\mu _{\gamma \gamma }$. On general grounds, a cancellation between the up-type and down-type contribution would be anyway difficult to realize since it would require a very strong deviation from the custodial symmetry limit, which is disfavored by EWPT.

3 This constraint on the Yukawa coupling can be relaxed by adding more families of VLF and/or considering higher values of *Y*. However, we shall not consider these cases throughout this work.

4 As already pointed out, the reader should take these expressions as illustrative. The contribution of the coefficient *b* is not necessarily negligible. We also recall that the velocity expansion is not valid in the presence of s-channel resonances (relevant in the 2HDM section), coannihilations and thresholds corresponding to the opening of annihilation channels [53].

5 For simplicity we have assumed that the t-channel diagrams are dominated by the exchange of the lightest mass eigenstate.

6 Our analysis is rather qualitative since based on one-loop $\beta$ functions. Our results are nevertheless in good agreement with the more detailed study presented in [58].

7 Since we are coupling the VLFs to both doublets, we cannot rigorously refer to type-I, type-II, lepton-specific or flipped 2HDMs, as flavor violating Yukawa couplings, possibly responsible for FCNCs, might be induced radiatively by the VLLs. We will nevertheless retain the classification of the various 2HDM realizations in order to distinguish the different dependence on $\tan \beta$ of the couplings of the SM fermions and the Higgs mass eigenstates.

8 Notice that even if the couplings $\lambda _6$ and $\lambda _7$ have been set to zero, they are radiatively generated. So one should also consider their $\beta$ function as well as additional terms in Eqs. (42)–(46). For simplicity we have not explicitly reported these contributions but we have included them in our numerical computations.

9 We remark that our discussion has mostly qualitative character since it is based on one-loop $\beta$-functions.

10 In the computation we have considered only a “perturbative” enhancement. A further enhancement can be achieved through non-perturbative effects [80], at the price of a rather strong fine tuning of $|m_A/2-m_{E_1}|$. We will not consider this case in the present work.

11 This requirement can be partially relaxed by introducing more than one family of VLL.

12 We have explicitly checked the other conditions (34) and (35) as a function of the energy and found that these are violated at a slightly lower scale of $5\times 10^{5}\,\text{ GeV }$. This difference is acceptable as it does not affect the validity of our results: our goal is not to quantitatively determine the scale at which the theory should be completed, but just to set a qualitative criteria that applies to the theory at low energy.

13 To a lower extent also the process $N_2 N_2 \rightarrow W^+ W^-$ can be relevant.

14 This possibility is contrived because the very strong *HAZ* coupling would easily lead to very high decay widths, which would make difficult the observation of resonances.

15 In fact, we checked that such a texture for the mass matrix suppresses, in a similar way as for $h\rightarrow \gamma \gamma$, the VLL contributions to $h\rightarrow Z\gamma$, thus leaving the latter decay SM-like.

16 Alternatively VLLs might originate with the decay of produced heavy neutral Higgses [102–104].
